# Structure and evolution of the squamate major histocompatibility complex as revealed by two *Anolis* lizard genomes

**DOI:** 10.3389/fgene.2022.979746

**Published:** 2022-11-08

**Authors:** Daren C. Card, Andrew G. Van Camp, Trenten Santonastaso, Michael I. Jensen-Seaman, Nicola M. Anthony, Scott V. Edwards

**Affiliations:** ^1^ Department of Organismic and Evolutionary Biology, Harvard University, Cambridge, MA, United States; ^2^ Museum of Comparative Zoology, Harvard University, Cambridge, MA, United States; ^3^ Department of Biological Sciences, University of New Orleans, New Orleans, LA, United States; ^4^ Department of Biological Sciences, Duquesne University, Pittsburgh, PA, United States

**Keywords:** BAC sequencing, comparative genomics, immunity, MHC, natural selection, squamata

## Abstract

The major histocompatibility complex (MHC) is an important genomic region for adaptive immunity and has long been studied in ecological and evolutionary contexts, such as disease resistance and mate and kin selection. The MHC has been investigated extensively in mammals and birds but far less so in squamate reptiles, the third major radiation of amniotes. We localized the core MHC genomic region in two squamate species, the green anole (*Anolis carolinensis*) and brown anole (*A. sagrei*), and provide the first detailed characterization of the squamate MHC, including the presence and ordering of known MHC genes in these species and comparative assessments of genomic structure and composition in MHC regions. We find that the *Anolis* MHC, located on chromosome 2 in both species, contains homologs of many previously-identified mammalian MHC genes in a single core MHC region. The repetitive element composition in anole MHC regions was similar to those observed in mammals but had important distinctions, such as higher proportions of DNA transposons. Moreover, longer introns and intergenic regions result in a much larger squamate MHC region (11.7 Mb and 24.6 Mb in the green and brown anole, respectively). Evolutionary analyses of MHC homologs of anoles and other representative amniotes uncovered generally monophyletic relationships between species-specific homologs and a loss of the peptide-binding domain exon 2 in one of two *mhc2β* gene homologs of each anole species. Signals of diversifying selection in each anole species was evident across codons of *mhc1*, many of which appear functionally relevant given known structures of this protein from the green anole, chicken, and human. Altogether, our investigation fills a major gap in understanding of amniote MHC diversity and evolution and provides an important foundation for future squamate-specific or vertebrate-wide investigations of the MHC.

## Introduction

The major histocompatibility complex (MHC) functions in adaptive and innate immunity against pathogenic infection. As such, the region containing MHC genes is one of the most variable and evolutionarily informative features of jawed vertebrate genomes. The MHC recognizes foreign proteins and presents them to immune cells by breaking these proteins down into small peptides and loading them onto specific molecules on the cell membrane. The cell membrane-bound protein complexes of the MHC are interrogated by circulating T-cell populations to trigger complex immune responses (Blum et al., 2013; Kaufman, 2018). The MHC therefore mediates both the innate and adaptive immune responses and protects the host from inter- and intra-cellular pathogens (Janeway et al., 2001a; Kelley et al., 2005).

The MHC region has many unique characteristics that play a role in diverse biological processes mediated by the genes in this region. Early studies of mammals suggested an archetypal model for the organization of the MHC region into three subregions based on the identities of the genes—the class I and II regions and the class III complement region. Genes within these regions are further distinguished into classical MHC molecules, which are highly polymorphic and function in the core process of binding and presenting peptides, and nonclassical MHC molecules, which show lower variation but still largely function in immunity (Beck and Trowsdale, 2000; Horton et al., 2004; Trowsdale and Knight, 2013; Kaufman, 2018). In the class I region, classical MHC genes encode the heavy (α) chains encoding class I MHC molecules, which are expressed on all nucleated cells and mainly present endogenous peptides to CD8^+^ T cells (Bjorkman and Parham, 1990). The class II region is characterized by paired A and B genes that encode the ⍺ and β chains of the classical class II MHC molecules, respectively. Classical class II molecules are expressed only on antigen-presenting cells like B cells or macrophages and present peptides to CD4^+^ T helper cells (Doherty and Zinkernagel, 1975). Nonclassical genes, many of which function generally in immunity and specifically in the complex pathways of biosynthesis, antigen degradation, translocation, and peptide loading that interact with classical MHC molecules, are found in both class I and II regions. Between these two regions in mammals is a class III, or complement, region with a variety of genes, including several involved in innate immunity (Janeway et al., 2001b).

High gene density and many gene duplications make MHC regions uniquely challenging to assemble and analyze relative to other regions of the genome (Dilthey et al., 2015; Dilthey, 2021). The peptide-binding regions of classical MHC genes are subject to strong balancing selection and can exhibit higher rates of non-synonymous evolution (Kelley et al., 2005; Shiina et al., 2006; Spurgin et al., 2011), which results in a high number of alleles, allowing the immune system to detect a wide range of pathogens (Gaudieri et al., 1997). As a result, the MHC region has the highest variation of any genomic region (Mungall et al., 2003) and the peptide binding region of the human leukocyte antigen class II (HLA2/MHC2) gene is the most polymorphic exon in the vertebrate genome (The MHC sequencing consortium, 1999; Hedrick, 2002). The MHC locus can evolve through a complex birth/death cycle in which a portion of the MHC may duplicate through ectopic recombination, diversify, or lose function through pseudogenization (Nei et al., 1997). This high copy number, in turn, drives changes in local patterns of genetic variation and phylogenetic relationships in MHC regions through gene conversion (Ohta, 1999; Chen et al., 2007; Hosomichi et al., 2008).

The structure of the MHC region often can vary greatly among major lineages of amniotes surveyed to date. Modest variation in the composition and arrangement of genes is evident across placental mammals (Kumánovics et al., 2003; Hurt et al., 2004; Dukkipati et al., 2006; Lunney et al., 2009; Ellis and Hammond, 2014; Abduriyim et al., 2019), and marsupials are characterized by several more differentiated MHC organizations (Belov et al., 2006; Siddle et al., 2011; Cheng et al., 2012), such as the relocation of class I genes away from the MHC class II and III regions in wallabies (Deakin et al., 2007; Siddle et al., 2009). However, mammal MHC regions are generally larger and contain more complex assemblages of genes than MHC regions in birds (Kelley et al., 2005). The first characterized MHC in birds, from chicken (*Gallus gallus*), was labeled as a “minimal essential MHC” due to its small size and simple architecture (Kaufman et al., 1999). Characterizations of MHCs in other avian lineages do not show such extreme minimal compositions (Hess and Edwards, 2002; Shiina et al., 2004; Balakrishnan et al., 2010; O’Connor et al., 2019; He et al., 2021, 2022), and although some avian MHCs are distributed across multiple chromosomes (Balakrishnan et al., 2010), birds in general have MHC regions that are more compact and contain smaller numbers of genes. Miller et al. (2015) characterized the MHC of the tuatara (*Sphenodon punctatus*), the sister lineage to Squamata, localizing the core MHC region to the q-arm of chromosome 13 and identifying seven class I and eleven class II MHC loci, including some copies on alternative autosomes and scaffolds that indicate multiple duplication and translocation events. This study reported that class I and class II genes are interspersed with each other, as is the case for the chicken and quail; however, the order and linkage associations of those genes remain unknown. The structure of the tuatara MHC was further explored as part of the characterization of its first reference genome (Gemmell et al., 2020). Fifty-six MHC genes spanning 13 scaffolds were identified, including six class I genes, six class II genes, 19 class III genes, 18 framework genes, and seven extended class II genes. The high gene content and complexity of the core tuatara MHC region resembles those of amphibians and mammals (Gemmell et al., 2020).

**FIGURE 1:**
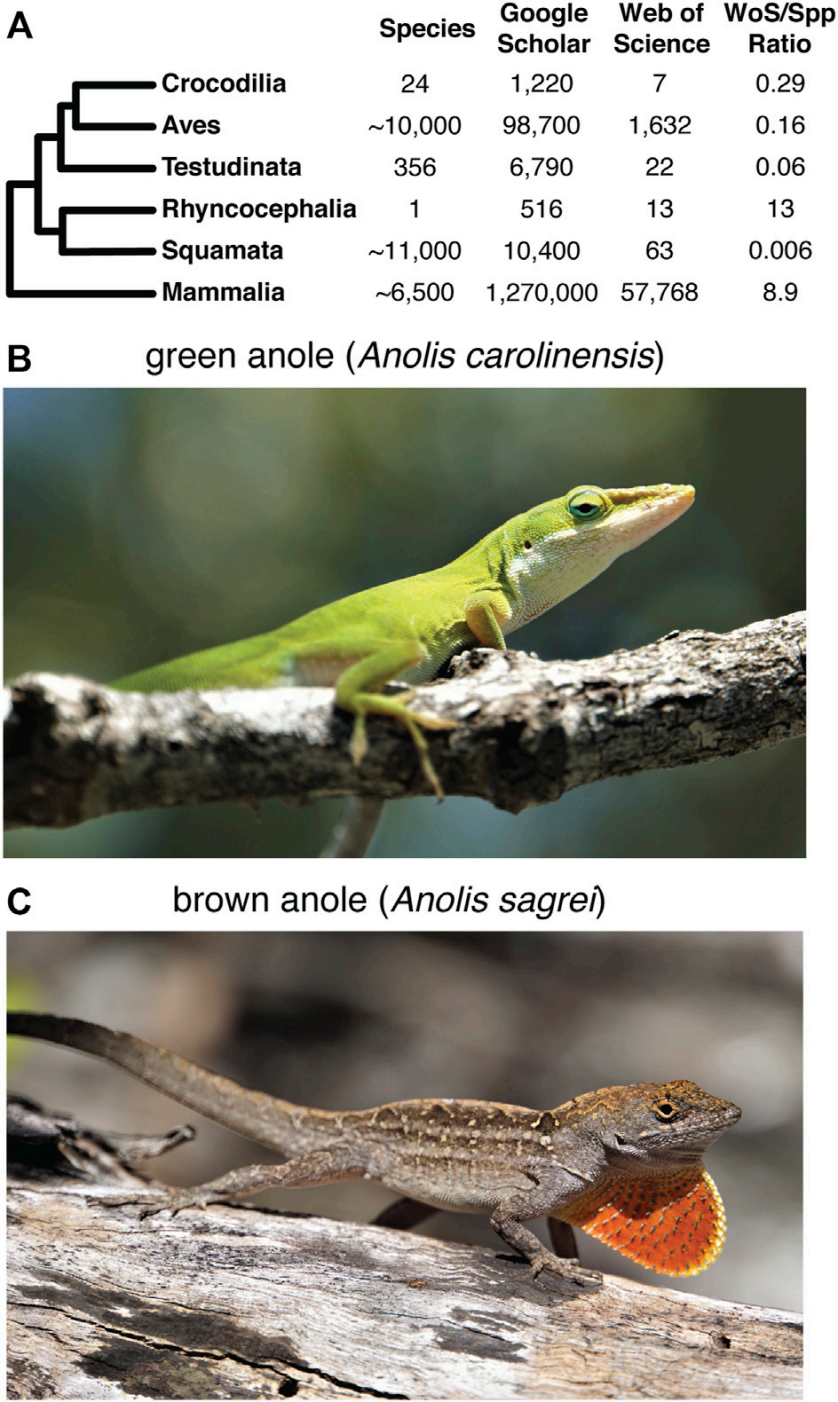
Overview of previous research on the squamate MHC and the focal species for this study. **(A)** Summary of the approximate number of species in each major amniote clade and the number of search results of literature searches of Google Scholar and Web of Science using keywords for MHC and the respective amniote clade. The ratio of the number of search result hits based on the Web of Science query to the approximate number of known species for each clade provides a conservative picture of the disparity in MHC research effort in each amniote lineage. See Supplementary Materials and Methods for more details of how the literature search was performed. The literature searches were performed on 10 March 2022. Photos of a **(B)** green anole (*Anolis carolinensis*) and **(C)** brown anole (*Anolis sagrei*). Photo credits: Nicholas Herrmann.

A detailed understanding of the structure and organization of the squamate MHC is notably absent from these comparisons. This deficiency likely stems from relatively little scientific interest in reptilian immunity and mate choice, and the paucity of reference genomes for squamate reptiles. However, numerous natural history traits shared between squamates and other vertebrates or unique to Squamata may have links to immunity and mate choice and, therefore, the MHC. The few studies of squamate MHC variation conducted thus far are frequently motivated to better understand the genetic consequences of reduced population sizes (Ujvari and Belov, 2011) and the biology of behavioral traits like sexual selection and mate choice (Bonneaud et al., 2006; Milinski, 2006; Piertney and Oliver, 2006). Although an unpublished dissertation did illuminate aspects of the green anole MHC (Godinez, 2012), only the MHC region of the Komodo dragon (*Varanus komodoensis*) has been generally characterized based on genome assemblies. This analysis identified an MHC with genes clustered into subregions, a broad organization similar to other non-mammalian taxa, and multiple copies of class Iα and class IIβ genes (Reed and Settlage, 2021). Otherwise, previous studies have largely focused on structure and variation of individual MHC genes of squamate reptiles. In Galápagos Marine Iguanas (*Amblyrhynchus cristatus*), *MHC I* genes possess conserved peptide-binding residues found in other vertebrates but lack the transmembrane and cytoplasmic domains necessary to anchor the class I receptor molecule into the cell membrane (Glaberman et al., 2008). The antigen-binding portion of *MHC I* genes in Squamata may also be subject to purifying selection instead of balancing selection, which has been detected in other vertebrate lineages (Glaberman et al., 2008). An investigation of MHC II in *A. cristatus* determined that all *MHC IIβ* homologs were monophyletic, suggesting that they evolved from a common ancestor after the divergence of squamates from other vertebrates and that the β-1 domain exhibits signatures of positive selection and interlocus gene conversion (Glaberman et al., 2009). Unfortunately, based on our literature search, there are relatively few studies that address the organization of the squamate MHC region, which leaves a significant gap in our understanding of MHC evolution in vertebrates, especially considering the high and growing diversity of squamate reptiles characterized taxonomically (Figure 1A). Thoroughly characterizing the structure of the MHC in squamate reptiles will provide a greater contextual framework for our understanding of MHC evolution in reptiles, and vertebrates in general.

Anoles have emerged as an important model research system (Losos, 1994; Lovern et al., 2004) and previous studies of the MHC in anoles have provided early insights into aspects of MHC biology in this species-rich clade. Hung et al. (Hung, 2013) documented high amounts of class I diversity in invasive brown anoles (*Anolis sagrei*), which could contribute to this species’ success in colonizing the southern United States. The crystal structure of the MHC I molecule in *Anolis carolinensis* was also recently solved and found to be unique among previous structures from other lineages (Wang et al., 2021). However, despite these studies of MHC in anoles and the status of the green anole as the first released squamate reference genome, no focused investigation of the composition and structure of the MHC region has been carried out in the green anole genome. Aside from solving the crystal structure of the MHC I molecule, Wang et al. (2021) also identified three classical MHC I loci and a β-2 microglobulin locus in the green anole genome. Moreover, Gemmell et al. (2020) performed a cursory annotation of the green anole MHC region for comparative purposes and found that the green anole MHC more closely resembles the MHCs of tuatara, mammals, and amphibians. Only a single class I gene was identified and no class II genes were found, suggesting the MHC region is incompletely assembled in the green anole genome. Our knowledge of MHC biology therefore remains rudimentary in both anoles and the broader, species-rich radiation of squamate reptiles.

To fill this gap, we investigated the structure and organization of the MHC of Squamata. *Anolis carolinensis* (Figure 1B), the first squamate to have its genome sequenced (Alföldi et al., 2011), is one of the few, and often only, squamate species included in most comparative genomics databases. *A. carolinensis* continues to be an important model system for understanding a variety of ecological and evolutionary questions (Losos, 1994), which justifies a detailed characterization of the green anole MHC. We also investigated the MHC of the brown anole (*A. sagrei*; divergence of approximately 39 million years from *A. carolinensis* based on the TimeTree database (Hedges et al., 2006; Kumar et al., 2017); Figure 1C) using a recent assembly generated for this species that is of higher quality than the existing green anole reference genome (Geneva et al., 2022). We characterized the composition and arrangement of classical and extended class I, II, and III genes within each anole species in relation to representative MHCs from other major amniote lineages. We hypothesized that a single, contiguous region would characterize the squamate MHC region as has been observed in mammalian and most avian genomes studied to date (but see Balakrishnan et al. (2010)).

## Materials and methods

### Identifying genomic regions with MHC similarity or homology from BAC sequencing

We screened a genomic BAC library (CHORI-318, BACPAC Genomics) derived from a male *A. carolinensis* from Dry Bay, Savannah River Site, Aiken County, South Carolina, United States (Alföldi et al., 2011). We identified candidate immune-related genes within the MHC complex by searching for homologs in GenBank (Sayers et al., 2021; assembly–AnoCar2.0, annotation release 101; GenBank accession–GCA_000090745.2) using key terms from 24 genes in the mammalian MHC region (see Supplementary Figure S4 and Supplementary Table S1). We used overlapping oligo (“overgo”) probes designed to map to exons within candidate MHC loci to screen nine high density membranes from the *A. carolinensis* BAC library (Supplementary Table S2). We retrieved all publicly available BAC end sequences (BES) from the Broad Institute corresponding to BACs that successfully hybridized with our overgo probes. We then searched the AnoCar2.0 assembly *via* BLAT on the UCSC genome browser for additional information on the genome assembly surrounding the overgo probe (Kent, 2002; Kent et al., 2002). We prioritized five BACs for sequencing based on patterns of hybridization with identified MHC genes and where at least one BES clearly flanked the probe and those that flanked gaps in the AnoCar2.0 assembly. BACs were sequenced at the Genomics and Proteomics Core Laboratories at the University of Pittsburgh at 60X coverage using a 454 FLX sequencer (Roche Diagnostics, IN). To determine the location of BACs, and thus MHC homology, in the *A. carolinensis* genome, we retrieved all BES sequences produced as part of the *A. carolinensis* genome sequencing project from the NCBI Trace Archive, along with the assembly data of these sequences from the Broad Institute’s FTP site. We also aligned contigs from the five sequenced BACs to the AnoCar2.0 assembly using BLAT.

### BLAST identification of genomic regions with MHC similarity

To identify putative MHC regions in the green and brown anole, we evaluated evidence for homology using 414 known MHC gene identifiers for human (*Homo sapiens*), mouse (*Mus musculus*), and chicken from the literature (The MHC sequencing consortium, 1999; Kelley et al., 2005; Supplementary Table S3). We searched GenBank using Entrez E-Utilities v. 16.2 (Brown et al., 2015) to identify proteins in Squamata using the query "{HGNC} [GENE] AND Squamata [ORGN]" where {HGNC} represents the human gene symbol for each gene. Given that squamate MHC proteins are less represented in Genbank, we also performed a similar search with MHC proteins from human and mouse using the query “{HGNC} [GENE] AND [*Homo sapiens* (ORGN) OR *Mus musculus* (ORGN)]”. Protein FASTA records for each hit were downloaded and used as queries to search for homology against each genome using BLAST v. 2.9.0+ (Altschul et al., 1990) with the tblastn algorithm and default parameters. We quantified the number of BLAST hits per scaffold and non-overlapping 100 kb windows on scaffolds greater than 1 Mb in length to identify genomic regions in each assembly with significant signal for homology with MHC genes.

### Placing and orienting MHC region scaffolds in the green anole

Our BLAST search yielded significant signals for MHC homology at the end of chromosome 2 for *Anolis carolinensis* and across several unplaced scaffolds. Given that MHC loci are generally located in a single genomic region in most species studied to date, including our analysis of *A. sagrei*, these patterns of MHC homology strongly suggest that the MHC region is poorly assembled in *A. carolinensis*. We used the improved contiguity of the brown anole genome to build a more complete assembly of the green anole MHC region under the assumption that this region of each genome is syntenic between anole species. We performed whole genome alignment between the green and brown anole genomes using Cactus v. 2.0.1 (Armstrong et al., 2020) with default settings. We also used two additional approaches to corroborate our inference of synteny and the placement and orientation of unplaced scaffolds based on our whole genome alignments. First, we leveraged spatial information available from RNA-seq data, where the distribution of exons across relatively large distances in the genome can be used for assembly scaffolding. Briefly, RNA-seq data for a variety of tissue types were downloaded from the Bgee database (Bastian et al., 2021) and we used AGOUTI v. 0.3.3-25-ga7e65d6 (Zhang et al., 2016) to further scaffold the green anole genome based upon these RNA-seq data. Second, we leveraged our BAC data to determine whether our BAC sequencing data spanned breakpoints between scaffolds in the existing green anole genome assembly. BES pairs that mapped to separate scaffolds were used to identify BACs putatively spanning multiple scaffolds whose sequences were mapped against the genome using BLAST to identify putatively syntenic scaffolds. Additional details of these analyses are included in the Supplementary Material.

### Manual identification of MHC genes

We performed a detailed manual annotation of protein-coding genes in each anole species and the genomes of human, chicken, zebra finch (*Taeniopygia guttata*), and tuatara. For known MHC genes for human, mouse, and chicken from the literature (The MHC sequencing consortium, 1999; Kelley et al., 2005), we searched each gene ID against the Ensembl database (Howe et al., 2021; release 105) for the green anole, human, chicken, zebra finch, and tuatara genomes. We cross-referenced the location of identified gene models with those locations that showed significant homology with MHC genes based on our BLAST-based localization analysis to reduce the probability of spurious information for gene identities or orthology in the Ensembl database. The brown anole reference genome was not yet included in Ensembl during this process, so we manually assessed gene identifiers in the annotation GTF file for the genomic regions with large amounts of MHC homology. Once we had produced a complete gene map of MHC genes for each species, we estimated the size of the MHC as the summed distance between the first and last annotated MHC gene on each MHC scaffold/chromosome.

We also attempted to estimate the total number of core *mhc1* and *mhc2* genes identified and published in the green and brown anole genome assemblies. To do so, we started with known *mhc1* and *mhc2* genes in human, mouse, and chicken and manually identified orthologous gene models for green anole, tuatara, and zebra finch using Ensembl release 105. For the brown anole, which was not yet included in Ensembl during this process, we used tblastx to search the genome for MHC CDS sequences from all squamates, tuatara, human, mouse, chicken, and zebra finch and cross-referenced these hits with gene models present in the brown anole gene annotation. Full details of the identification of MHC genes are included in the Supplementary Material.

### Comparative characterization of MHC regions

We assessed both genome-wide and MHC-specific patterns of several genomic features for both anole species, tuatara, chicken, zebra finch, and human. Repeat element compositions are known to turn over significantly between major clades of vertebrates and local patterns of repeat composition in genomes can be variable. Human repetitive content was quantified using RepeatMasker v. 4.1.2-p1 (Smit et al., 2013) using human repeat consensus sequences from Repbase v. 3.3 (2020-11-09; Jurka et al., 2005; Bao et al., 2015). For the chicken and zebra finch genomes, we used RepeatMasker to quantify repeats based on consensus sequences for the clade Aves. Because Lepidosauromorpha repeats are less represented in Repbase, we first generated a *de novo* repeat library for the tuatara and both anole genomes using RepeatModeler v. 2.0.2 (Flynn et al., 2020) with default settings. We annotated repeats in the tuatara and both anole genomes using RepeatMasker, first using vertebrate repeat consensus sequences from Repbase and then using the appropriate species-specific *de novo* repeat library.

For each annotated gene of each species, we used tabix v. 0.2.5 (Li, 2011), bedtools v. 2.29.0 (Quinlan and Hall, 2010), and custom scripts to quantify the numbers and lengths of intron and exon gene features and to measure intergenic distances. To understand patterns of GC content in MHC regions, we estimated GC content at third codon positions (GC3) for each annotated gene for all species using gffread v. 0.12.1 (Pertea and Pertea, 2020), seqkit 2.1.0 (Shen et al., 2016), and custom scripts. We used ggstatsplot v. 0.9.1 (Patil, 2021) in R v. 4.0.3 to statistically compare the distributions of repeat content, intron lengths, intergenic distances, and GC3 content in MHC regions *versus* the entire genome across all species. Distributions were compared using a nonparametric Kruskal–Wallis one-way ANOVA test with pairwise comparisons inferred using *post hoc* Dunn tests, where *p*-values were corrected for multiple comparisons across all pairwise comparisons using the Bonferroni correction method (Dunn, 1961).

### Estimating gene orthology in MHC regions

We evaluated the homologous relationships between genes in the green anole, tuatara, chicken, zebra finch, and human MHC regions using the Ensembl comparative genomics database release 105 (Howe et al., 2021), which classifies homology into one of three categories based on species relationships and gene duplications. 1-to-1 orthologs describe homologous, single-copy genes in all species compared, and 1-to-many or many-to-many orthologs reflect instances where the number of homologs in one or more compared species is greater than one due to duplications or losses in one or more species. We queried 113 known human MHC genes and gathered all orthologs inferred in the four other species.

### Estimating squamate MHC gene family evolutionary histories

We built alignments of relevant exons for *mhc1* and *mhc2β* genes as a means of inferring patterns of evolution of these critical immune molecules. As described above, we used known MHC genes from human, mouse, and chicken and Ensembl release 105 to identify putative *mhc1* and *mhc2β* homologs in green and brown anoles, zebra finch, tuatara, and six other squamate species. For each putative MHC gene, we extracted CDS sequences for all gene transcripts for all squamate species, tuatara, chicken, zebra finch, mouse, and human. For the brown anole, we used the results of a tblastx search (described above) to identify putative homologs and exon sequences for all gene models were extracted for each putative *mhc1* and *mhc2β* homolog in the brown anole genome using gffread v. 0.12.1 (Pertea and Pertea 2020). Separately for *mhc1* and *mhc2β* homologs, we used MAFFT v. 7.407 (Katoh and Standley 2013; Katoh et al., 2005) with the options-auto and-adjustdirectionaccurately to produce an initial alignment. We refined each alignment by removing homologs that aligned poorly or which had large amounts of missing data, realigning portions by codon using TranslatorX (Abascal et al., 2010), manually adjusting portions of the alignment, and verifying intact ORFs for each exon.

We used a concatenation of exons 2 and 3 for both *mhc1* and *mhc2β* to produce phylogenies for each gene across all 13 amniotes (although for class II, no genes for *Laticauda laticauda* and *Naja naja* passed our stringent criteria for inclusion in the alignment). Phylogenies were estimated using the maximum likelihood method as implemented in IQ-Tree v. 2.1.4-beta COVID-edition (Minh et al., 2020). We used the model selection tool ModelFinder (Kalyaanamoorthy et al., 2017) to find the best substitution model. To model the evolutionary history of MHC gene family size for both class I and II, we took the total numbers of genes in each class for each species (separately and combined) and analyzed these tables using the software Bayesian Estimation for Gene Family Evolution (BEGFE) v. 2.0 (Liu et al., 2011) using default parameters and a variable rate of birth-death (lambda, λ). This method estimates the probability of gene family numbers expanding, contracting, or remaining constant on each node of the species tree. We ran the MCMC chain for 10 million steps, sampling every 1,000 steps. For these analyses we used the rooted species tree of the 13 species, including those for which 0 genes were recorded. We evaluated the posterior distributions of the estimated parameters in Tracer (Rambaut et al., 2018).

### Measuring natural selection in MHC genes

We first used omegaMap v. 0.5 (Wilson and McVean, 2006) to infer natural selection across codons of all exons of *mhc1* and *mhc2β* in one or both *Anolis* species. We ran omegaMap separately on *mhc1* alignments for the green and brown anole and on the combined anole *mhc2β* alignment. We ran one million iterations, sampling every cycle and with a burn-in of 100 samples, using inverse or improper inverse priors as appropriate on the following parameters: mu (mutation rate), omega (dn/ds), kappa (transition/transversion ratio), rho (recombination rate), and indel (insertion/deletion rate). We modeled the parameter omegaParam using 0.01 and 100 to specify the distribution. We also studied natural selection using the Fixed Effects Likelihood module on the DataMonkey web server (Weaver et al., 2018) using HyPhy v. 2.1 (Kosakovsky Pond et al., 2005, 2020) on the same three alignment datasets used in the omegaMap analysis, plus an additional *mhc2β* alignment with a single *Podarcis muralis* homolog included as an outgroup. We used a default *p*-value threshold of 0.1 and selected all branches of gene trees for analysis throughout. For *mhc2β*, we retained only the full-length homologs that include exon 2 for both species, resulting in only a single homolog per species. Given the low number of anole homologs for *mhc2β*, we produced a combined alignment of homologs from both anole species, removing gap-only codons.

## Results

### Genome assembly assessments

As reported in Geneva et al. (2022), the new assembly of the brown anole genome is higher quality than the existing green anole reference genome. The brown anole genome is significantly more contiguous than the green anole genome (scaffold N50 of 253.6 Mb vs 150.6 Mb, respectively) and contains more complete, single-copy vertebrate BUSCOs (95.3% vs 87.2%, respectively; Geneva et al., 2022). We assessed the genome-wide repetitiveness of 27-mers across the core MHC region of each anole genome (see below and Supplementary Material) and found similar patterns in each species: k-mer depth varied from one to greater than 1,000 and most high depth k-mers were annotated as repetitive elements (Supplementary Figure S2). High depth 27-mers that were not annotated as repetitive elements may indicate assembly artifacts, such as falsely retained haplotypes, but are also expected in repetitive genomic regions with complex patterns of gene family evolution like in the MHC.

### BAC-based characterization of MHC in the *A. carolinensis* genome

**FIGURE 2:**
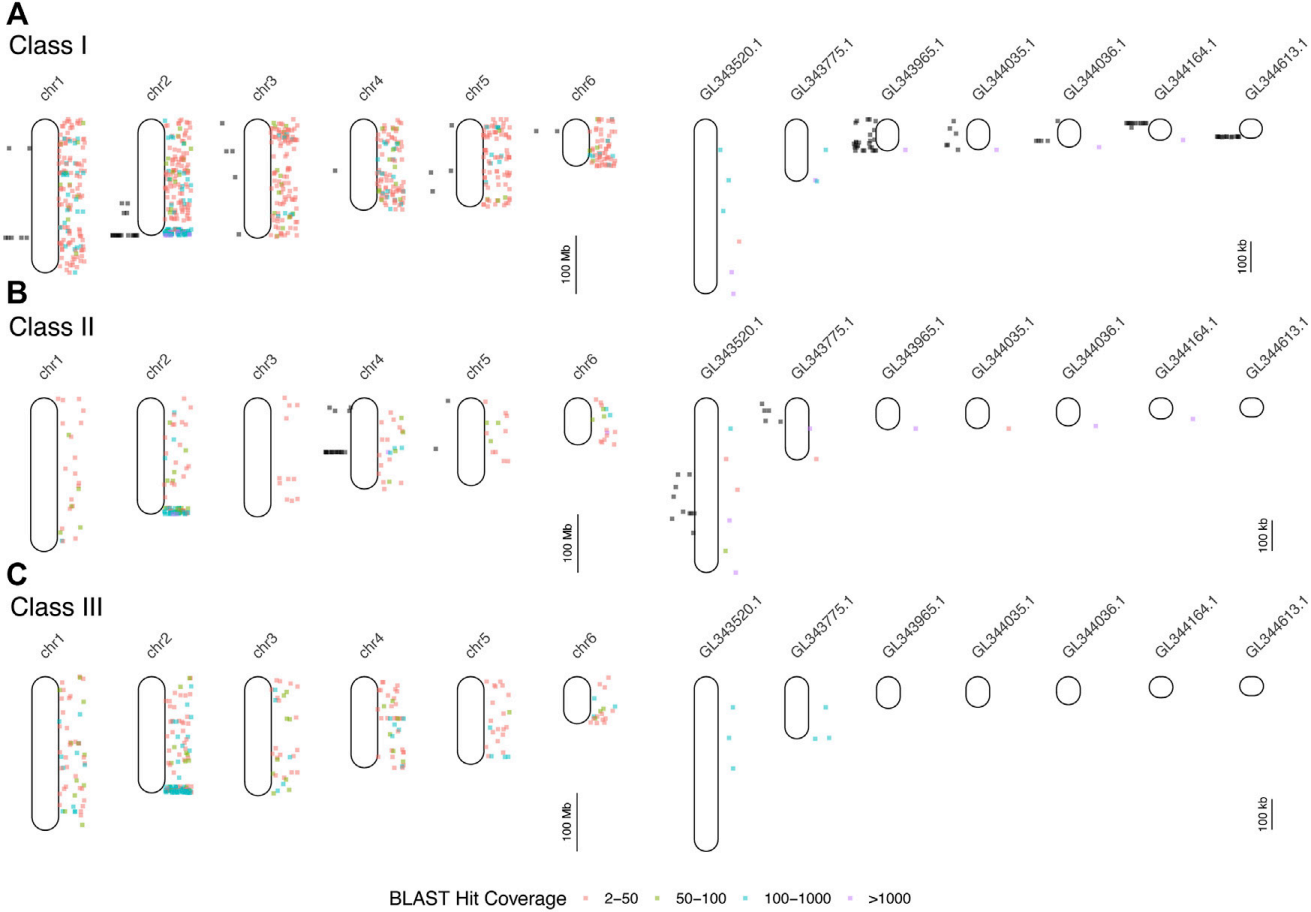
Results of mapping of BAC end sequences (BESs) of BACs detected in hybridizations (black dots to the left of each chromosome or scaffold) and BLAST searches of human and mouse MHC protein data to the green anole genome (colored dots to the right). Each point indicates the locations of BLAST hits (binned by hit coverage). Results are displayed separately for class I **(A)**, class II **(B)**, and class III **(C)** genes for all six chromosomes and for unplaced scaffolds with five or more mapped BESs. Note differences in scale between panels devoted to chromosomes (left column) and unplaced scaffolds (right column). See Supplementary Table S6 for the results of the BLAST mapping of BESs.

We searched GenBank for 24 MHC-related genes and retrieved a total of 334 loci from 67 vertebrate species (Supplementary Table S1). Comparing each of these 334 sequences to the AnoCar2.0 assembly using BLAT identified 87 unique loci, derived from 15 of the initial 24 MHC-related genes used as queries (vertebrate homologs from nine genes failed to find a significant match in AnoCar2.0; Supplementary Table S4). From these 87 loci, we designed 26 overgo probes and conducted a total of 18 hybridizations, resulting in 237 hits to the *A. carolinensis* BAC library CHORI-318 (Supplementary Table S5). The number of unique positive hybridizations, in parentheses, per gene were as follows: *mhc1* (157), *mhc2* (36), *tap2* (15); *tnxb* (6); *lmp2* (4); *lmp7* (4); *rxrb* (7); *blec1* (5); *ring1* (3). Since each hybridization was against 4-5X fold coverage of the genome, the last six genes in this list likely exist as single-copy genes; by contrast, *mhc1*, *mhc2*, and *tap2* appear to be found as multicopy genes and/or pseudogenes. The 237 BACs identified in the hybridizations cluster to several discrete locations in the genome, including *mhc1*-containing BACs on chromosomes 1 and 2, along with several unplaced scaffolds (Figure 2). BACs that hybridized to *mhc2* probes map to chromosome 4 and two unplaced scaffolds. There appears to be very little overlap in genomic location of *mhc1-*and *mhc2-*containing BACs, indicating that these two loci are not adjacent as they are in mammals. Assembly of individual sequence reads resulted in multiple contigs within each of the five BACs chosen for sequencing and an average N50 of about 23 kb (Supplementary Table S4). The remaining 48 (84.6%) BAC contigs at least partially aligned confidently to a conservative estimate of 15 genomic scaffolds and two chromosomes (Supplementary Table S4; Supplementary Figure S3). We resolved two large gaps in the overall AnoCar2.0 genome assembly with these sequence data. The alignment of BAC 107B15 with AnoCar2.0 reveals an omission of 4,289 bp between positions 20,281 and 20,282 in the genomic scaffold Un_AAWZ02036018. Secondly, BAC 134P17 contains a 9,812 bp sequence that completely covers a ∼10 kb gap in scaffold Un_GL343520 between positions 332,815 and 322,724. Importantly, this gap contains the coding sequence for a putative *mhc2* gene. See the Supplementary Material for a summary of each BAC assembly, the locations these contigs aligned to in the green anole genome, and the details of gene annotation of BAC assembly contigs.

### BLAST localization of *Anolis* MHC regions

**FIGURE 3:**
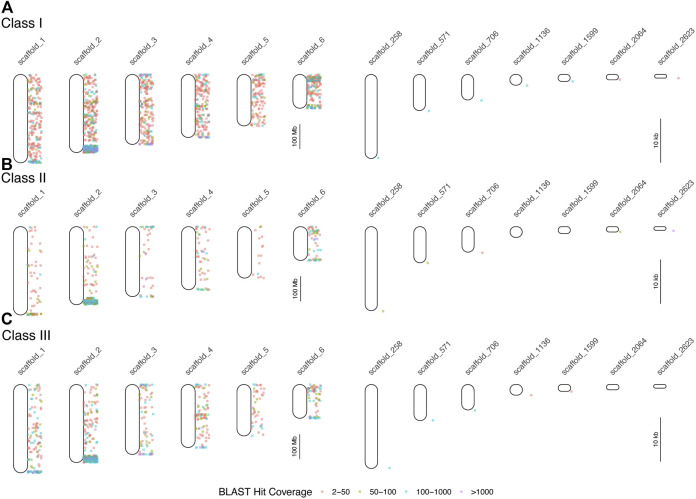
Results of BLAST searches of human and mouse MHC protein data in the brown anole genome. Each point indicates the locations of BLAST hits (binned by hit coverage; right of chromosome maps). Results are displayed separately for class I **(A)**, class II **(B)**, and class III **(C)** genes for all six scaffolds, which correspond to the six macrochromosomes, and for unplaced scaffolds with at least 100 hits and average hits per 100 bp of greater than 4. Note differences in scale between panels the panels devoted to chromosomes (left column) and unplaced scaffolds (right column).

We queried GenBank for 414 MHC-related protein identifiers (Supplementary Table S3) and retrieved 1,587 proteins from 26 squamate species, 63,828 human proteins, and 3,033 murine proteins. We searched for each of these proteins in the green and brown anole genomes using BLAST, which yielded 401,401 and 471,292 hits from squamate-derived proteins, respectively, and 3,417,387 and 2,693,796 hits from mammal-derived proteins, respectively. For both species, based on our BLAST search of human- or mouse-derived proteins, each set of hits localized MHC signal similarly, with significant signal for MHC homology observed on chromosome 2 (green anole) or scaffold 2 (brown anole) and appreciable amounts of MHC homology on chromosomes/scaffolds 1, 3, and 4 in each anole genome (Figure 3). Based on our BLAST search, 2,397,646 class I hits, 259,599 class II hits, and 183,818 class III hits were detected on unplaced scaffolds in the green anole genome (Supplementary Figure S6). In the brown anole genome, our BLAST search identified 2,081,003 class I hits, 348,203 class II hits, and 240,385 class III hits on scaffolds shorter than 1 Mb (i.e., putative unplaced scaffolds; Supplementary Figure S6). Sliding window counts of BLAST hits across the longest scaffolds in each species detected largely dispersed signal for MHC homology and the highest density of MHC homology in each genome, by far, was one end of chromosome 2 or scaffold 2, strongly suggesting this region contains the core MHC region in anole species (Figures 2, 3 and Supplementary Figures S4-S7). Similar qualitative patterns of homology were detected when using proteins from various squamate species to perform the BLAST search against each genome (Supplementary Figures S4-S7). Quantitative analysis of the BLAST results from the query using human and mouse proteins indicated that there were many more “off target” hits outside the identified MHC region than from the results of the query using squamate proteins. These artifacts are due to large biases inherent between the human/mouse or squamate proteins available from NCBI that were used to perform the BLAST searches (Supplementary Figure S8). For the results of the BLAST search using squamate proteins, we observed that most hits were for the genes *ZBTB12* and *BTN2A1*, which are both part of large gene families with diverse functions located throughout the genome (Afrache et al., 2012; Siggs and Beutler, 2012). This pattern was in contrast to the results from the BLAST search using human/mouse proteins, where high proportions of hits came from various MHC/HLA gene orthologs located predominantly in the MHC region.

### Placing and orienting MHC scaffolds in the green anole genome

**FIGURE 4:**
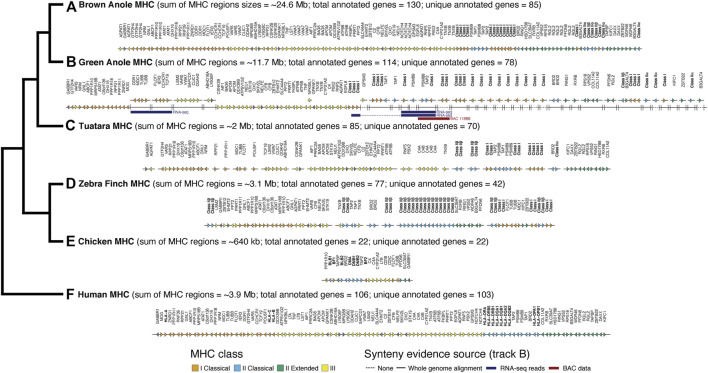
Comparative map of genes identified in MHC regions of six amniotes: **(A)** brown anole, **(B)** green anole, **(C)** tuatara, **(D)** zebra finch, **(E)** chicken, and **(F)** human. The total approximate size (sum of region sizes across scaffolds) and total and unique number of annotated genes in these regions for each species are indicated in parentheses. The estimated sizes of the MHC regions reflect the summed distances between the first and last annotated MHC gene on each chromosome/scaffold containing MHC genes. The phylogeny to the left shows the evolutionary relationships between each species. For green anole, we used whole-genome alignments and secondary evidence from BAC and RNA-seq data to infer the placement of scaffolds in the incompletely-assembled MHC region. We use a solid or broken line to indicate scaffolds where placement was or was not confidently inferred based on the whole genome alignments. Where placement was not confidently inferred, scaffolds were arranged based on the ordering of genes reflected in other species where the MHC regions were well assembled. RNA-seq reads (various read depths) and BAC data spanning multiple scaffolds, which provides increased confidence for the placement and orientation of scaffolds, are also indicated using blue (RNA-seq) and red (BAC) bars. For tuatara and zebra finch, we roughly ordered any scaffolds with MHC genes based on ordering in the other species. Importantly, the absolute gene direction (i.e., strand) is not confidently inferred between scaffolds for species where the MHC is assembled on multiple scaffolds. Note that our MHC annotations for the well-studied human and chicken genomes are missing some genes annotated in previous, more detailed studies of the MHCs of those species. See Supplementary Table S9 for a full summary of the chromosomal locations of MHC genes in each species and Supplementary Figure S9 for a graphical summary of the arrangement of green anole scaffolds in the MHC region based on the whole genome alignment with the brown anole genome.

Using the results of our whole genome alignment between the green and brown anole genomes, we were able to place 19 previously unplaced green anole MHC scaffolds in poorly assembled portions of chromosome 2 (Figure 4; Supplementary Figure S9; Supplementary Table S9). Four scaffolds fall at the boundary between class I and class II regions of the MHC and several large gaps in that region, including one that is 58 kb, suggest that this region is poorly assembled. The existing green anole chromosome 2 assembly ends at approximately the boundary between class III and class II regions of the MHC, suggesting that the complex, repetitive features of this region hampered assembly. The remaining 15 newly placed scaffolds fall after the existing chromosome 2 terminates and comprise most of the class II region (Figure 4; Supplementary Figure S9; Supplementary Table S9). Moreover, 15 additional scaffolds, most of which contained class I or class II genes, were also tentatively placed in this extended chromosome 2 region based on the presence of MHC genes contained within the brown anole MHC region. Further sequencing will be necessary to place these 15 scaffolds with greater confidence and derive a complete assembly of the MHC region of the green anole.

We were able to confirm the placement of a subset of scaffolds using alternative information derived from RNA-seq and BAC data. Based on multi-tissue RNA-seq dataset, we were able to scaffold 923 contigs/scaffolds in the original green anole reference genome. In the MHC region of the green anole, we found three pieces of transcriptomic evidence that spanned existing scaffolds (Ensembl/NCBI scaffold identifiers): 1) one transcript that unites GL343438/NW_003338985 and GL343623/NW_003339170; 2) one transcript that unites GL343748/NW_003339295, AAWZ02039800/NW_003343001, and GL344036/NW_003339585; and 3) one transcript that unites chromosome 2/NC_014777, GL343748/NW_003339295, AAWZ02039800/NW_003343001, and GL344036/NW_003339585 (see Figure 4B RNA-seq). We also evaluated evidence from our BAC data that spanned assembly gaps or enabled cross-scaffold orientation and placement. In addition to the gaps we closed noted above, BES and contig assembles for one BAC (118B8) spanned multiple MHC-region scaffolds: GL344036/NW_003339585 and GL344134/NW_003339687 (see Figure 4B BAC 118B8). A comparison of the class II region of the brown anole with this better-assembled version of the class II region for the green anole indicates that this region of the green anole genome remains poorly defined and future studies will be necessary to improve our understanding of the green anole MHC.

### Comparative summary of MHC region gene maps in mammals, birds, and squamates

After placing and orienting green anole scaffolds, we were able to summarize the extent, composition, and arrangement of genes in MHC regions for six amniotes, including the two anole species and human, tuatara, chicken, and zebra finch. Using MHC gene queries and the Ensembl toolkit, we estimated the size of the MHC for each species as the distance between the first and last annotated MHC gene. We found the MHC region of birds to be relatively compact, encompassing approximately 3 Mb in the zebra finch and only roughly 650 kb in the chicken. Mammals, on the other hand, are known to have larger MHC regions than birds; our estimate of the human MHC size was 3.9 Mb. Our results uncover an MHC region of about 2 Mb in tuatara, though the fragmentary assembly of this region may confound our estimation of the MHC margins and length in this species. In contrast, the MHC regions of both anole species were far larger at about 10.5 Mb and 24.5 Mb in the green and brown anole, respectively (Figure 4). Similar relative MHC sizes were evident in the fully assembled human and brown anole MHC regions when the genes *GABBR1* and *DAXX* were used as markers for the beginning and end of the MHC region (3.8 and 24.1 Mb MHC sizes, respectively).

The composition and arrangement of genes in the MHC regions of mammals and birds also differ. Mammals are characterized by a gene-rich MHC, and we were able to identify 103 of the approximately 260 annotated genes in the human MHC region in other species (Trowsdale and Knight, 2013). We identified 22 genes in the MHC region of chicken, which is fewer than the 45 genes previously reported (Shiina et al., 2007; Fulton et al., 2016), and 42 genes in the MHC region of zebra finch, which is greater than the previously reported count of 27 genes reported in the literature (Balakrishnan et al., 2010; Ekblom et al., 2011), though the zebra finch MHC region had not been fully characterized (Figure 4). Gene composition of the tuatara MHC is intermediate between mammals (as characterized in humans) and birds, with a previous investigation reporting 56 genes (Gemmell et al., 2020), fewer than the 70 genes identified in this study. Based on our analysis of the green and brown anole genomes, squamate MHC regions resemble the mammalian MHC by containing a relatively complete set of canonical extended MHC genes: 78 genes in the green anole and 85 genes in the brown anole (Figure 4). However, some genes found in the human MHC appeared absent in the MHC regions of both anole species. *LTA*, *LTB*, and *C4B*, all involved in innate immunity, were missing in both anole genomes and several additional genes were missing in individual anole genomes (see Supplementary Material), though assembly or annotations artifacts could contribute to this pattern. However, tblastx searches of each genome using human orthologs of these three genes only uncovered results for *C4B* (Supplementary Table S10), suggesting potential pseudogenization or that this gene may be present in the genome but has remained unannotated. Gene duplications were also evident, although misassemblies or other genomic artifacts could be driving these patterns. Numerous genes (e.g., *DDR1*, *GTF2H4*, *TNXB*, and *VWA7*) were duplicated in the anoles, especially in the brown anole, relative to the human. Notably, *AGPAT1* and *RPP21* were both duplicated in the brown anole genome and dispersed across the MHC region.

We observed numerous copies of both class I and II MHC genes in the genome assemblies of both anole species. At least 16 and 26 class I genes and six and four class II genes were identified in the brown and green anole genome assemblies, respectively. The relative proportions of class I and II genes identified in the green anole assembly (∼6 times as many class I as class II genes) is roughly similar to the numbers of positive BAC hybridizations observed for class I (157) and class II (36) genes in the green anole, suggesting we have identified most class I and II genes in this species. However, only a subset of class I and II genes appeared to have been sampled as part of our BAC sequencing, with only one class I gene and five class II genes annotated on assembled BAC contigs (see Supplementary Material for additional details of gene annotations on BAC contigs).

The arrangement and clustering of genes into distinct MHC regions observed in mammals and birds is also present in squamate reptiles, although certain genes have alternative arrangements in these different clades. Comparing the human and brown anole MHC regions, which are well assembled and gene-rich in both species, we found that genes generally clustered into regions based on MHC class in both species but with substantial rearrangements of genes between these two species. Several class I genes were located in the class II MHC region in both anole species, a pattern observed in all amniote clades studied to date except placental mammals. Among well-assembled portions of the green anole MHC, we also found evidence for alternative arrangements of some genes between the two anole species, especially in the class III region, where gene ordering in both species suggests one or more inversions in one or both anole species (Figure 4). Finally, we found evidence for the relocation of the *DMA* ortholog of the class II alpha chain away from the core MHC region to chromosome 4 in both anole species. In the green anole genome, the *DMA* gene (green anole Ensembl ID ENSACAG00000003989) has 1-to-1 orthology with the *DMA* ortholog from chicken and is located at coordinates 93,358,586–93,371,447 on chromosome 4. In the brown anole genome, we observed a single *DMA* homolog on chromosome 4 (coordinates 101,436,934 to 101,445,957), which was annotated as the *DMA* ortholog based on homology searches against the RefSeq database. This translocation likely occurred before the anole radiation and accounts for some of the signal of homology observed on chromosome 4 in both the BAC and BLAST-based localization of the MHC.

### Repeat content, intronic, and intergenic distributions in *Anolis* MHC regions

**FIGURE 5:**
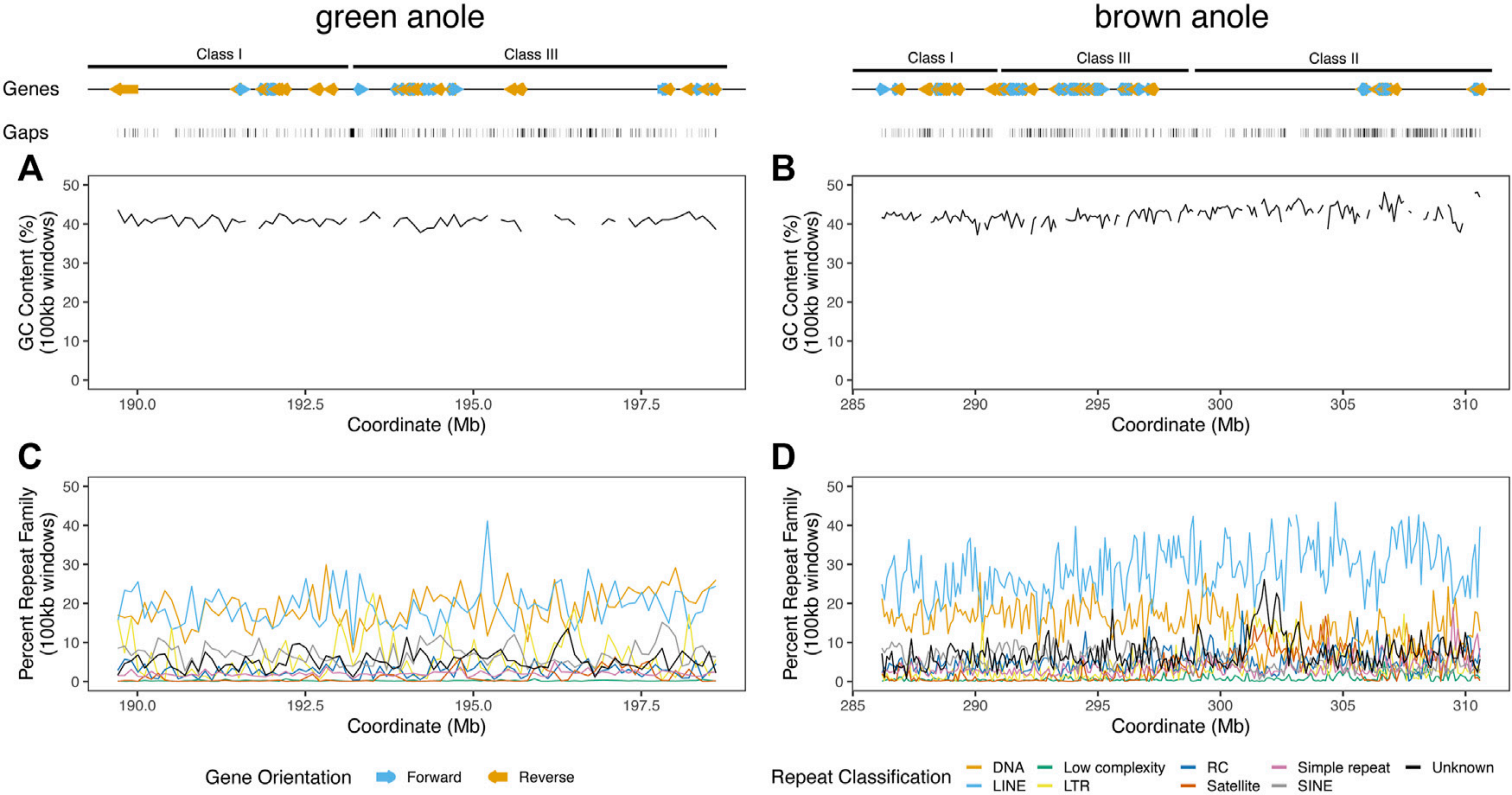
Landscapes of genomic features within the well-assembled portions of the core MHC regions in the green and brown anole genomes. GC percentage across non-overlapping 100 kb windows of the MHC regions of the **(A)** green and **(B)** brown anole genomes. Abundances of repeat elements in the **(C)** green and **(D)** brown anole MHC regions. Repeat abundances were quantified as the percentage of a given repeat classification in non-overlapping 100 kb windows. When the proportion of gaps (i.e., Ns) was greater than 10% in a window, we did not calculate GC percentage to avoid large fluctuations in GC content that are due to large amounts of missing data in a window. Protein-coding genes annotated (color and orientation aligns with gene orientation) and assembly gaps are displayed as tracks above the distributions of GC and repeat content. Abbreviations for repeat elements are as follows: DNA = DNA transposons, LINE = Long Interspersed Nuclear Element; LTR = Long Terminal Repeat elements; RC = Rolling Circle elements; and SINE = Short Interspersed Nuclear Element.

We supplemented our annotation of protein-coding genes with detailed characteristics of repetitive content and other features known to turn over between genomic regions or amniote lineages. In the MHC regions of the green and brown anole genomes, we found that genes clustered into gene islands separated by intergenic regions and assembly gaps and were evenly spread over the entire region (Figure 5). GC content in 100 kb windows was largely stable at approximately 40% for both species, but some variation was evident over the region and windows with the largest fluctuations in GC content had large proportions of gaps, indicating that measures of GC content are influenced by assembly gaps. Repeat element density across 100 kb windows was far more variable than GC content, with some windows showing marked shifts in repeat element abundance from the region-wide means of each species. Repeat compositions of MHC regions for both anoles were broadly similar, though the brown anole had higher abundances of LINE elements that generally grew across the MHC region, especially for windows beyond the 300 Mb coordinates (Figure 5; see Supplementary Material for more details).

**FIGURE 6:**
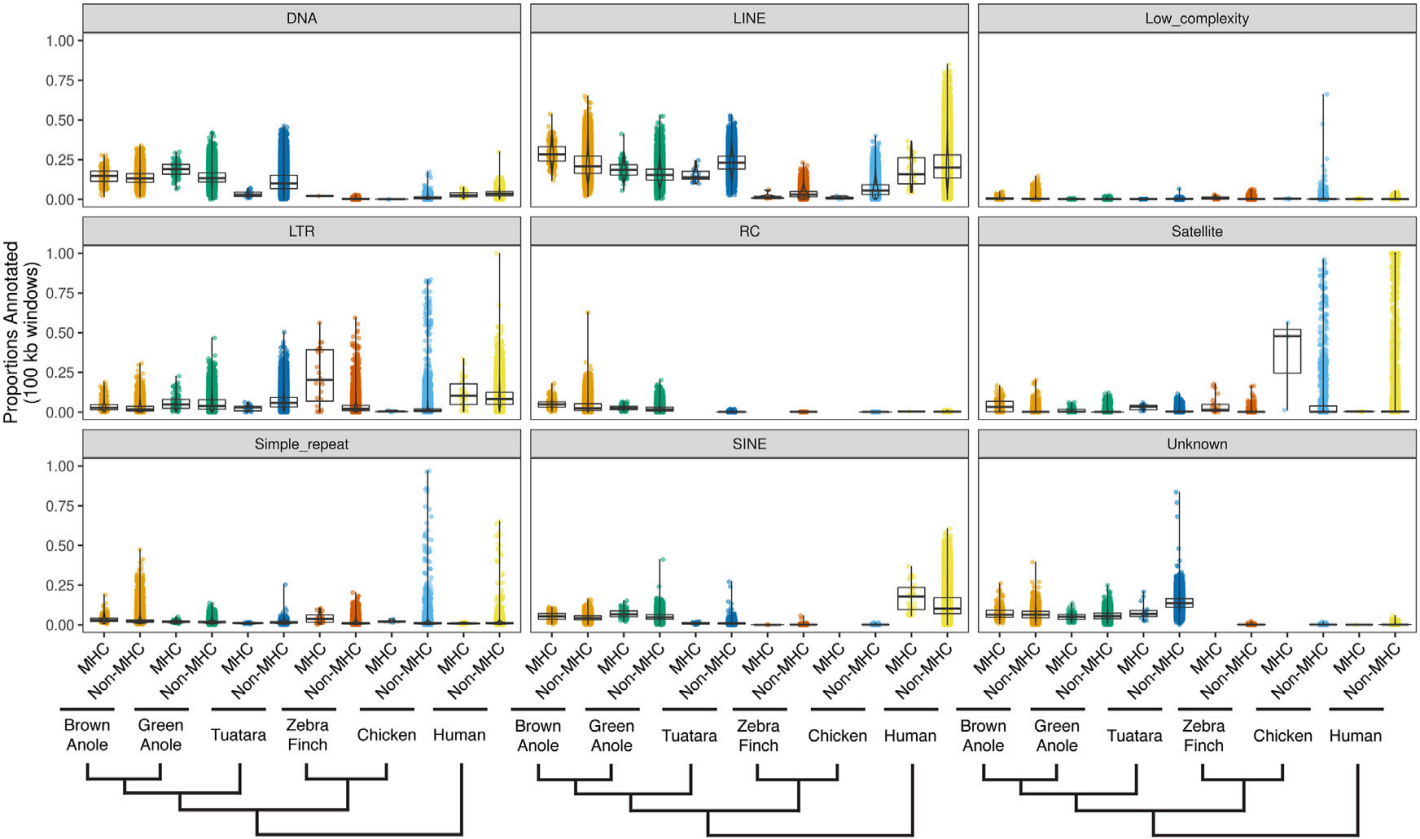
Quantitative summary of repeat element compositions across MHC and non-MHC regions of six amniote species. For each of nine major classifications of repeat elements, per-species distributions of the proportions of non-overlapping 100 kb windows were calculated for MHC and non-MHC regions. Species are arranged phylogenetically along the *x*-axis with a phylogeny that summarizes their evolutionary relationships. See Supplementary Figure S10 for the results of pairwise statistical comparisons between each repeat classification, genomic region (MHC and non-MHC), and amniote species. See Supplementary Table S11 for the summary statistics underlying repeat proportion distributions and the results of pairwise statistical comparisons between each repeat classification, genomic region (MHC and non-MHC), and amniote species. Abbreviations for repeat elements are as follows: DNA = DNA transposons, LINE = Long Interspersed Nuclear Element; LTR = Long Terminal Repeat elements; RC = Rolling Circle elements; and SINE = Short Interspersed Nuclear Element.

We evaluated patterns of repeat composition in MHC and non-MHC regions of the six amniote species. Across nine major repeat classifications, including unknown repeats, we detected significant differences between genomic regions and species (Figure 6; Supplementary Figure S10; $\chi_{Kruskal-Wallis}^{2}\left(101\right)=7.8\times 10^{5},\;p\leq 0.001$ ). LINE elements were appreciably abundant in the genomes and MHC regions of both anole species, tuatara, and human, although proportions of LINEs in MHC regions of squamates were higher than proportions in non-MHC regions (median proportions of LINEs in the MHC/non-MHC regions: green anole = 0.19/0.15 and brown anole = 0.28/0.21), which is the reverse pattern seen in tuatara and human, where MHC regions had lower proportions of LINE elements (median proportions of LINEs in the MHC/non-MHC regions: tuatara = 0.14/0.23 and human = 0.16/0.20). We also found that DNA transposons are relatively highly abundant in lepidosaur genomes. Unlike tuatara, where DNA transposons are far less abundant in MHC regions relative to the rest of the genome (median proportions of DNA transposons of 0.03 vs 0.1, respectively), DNA transposons in the two squamate species account for greater relative proportions of MHC regions (median proportions of DNA transposons in the MHC/non-MHC regions: green anole = 0.19/0.13 and brown anole = 0.15/0.13; Figure 6). The unique pattern of higher proportions of some repetitive content in squamate MHC *versus* non-MHC regions suggests a distinct evolutionary history for MHC regions in squamates. However, the large differences in the size of the MHC region described above may also contribute to this pattern because it results in differences in the number of genomic windows that underlie our comparisons of repeat content.

**FIGURE 7:**
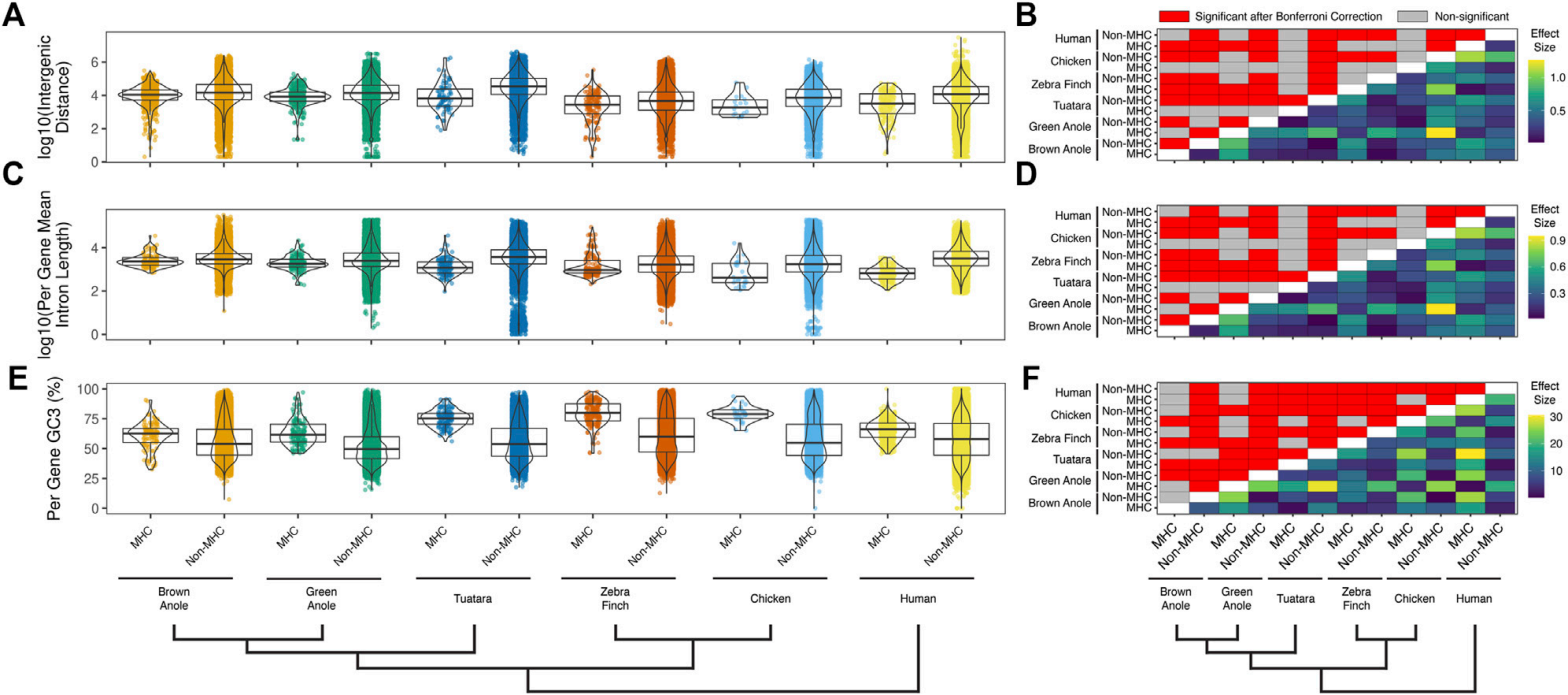
Quantitative comparisons of features of genomic composition in MHC vs non-MHC regions of six amniote species. Distributions of **(A)** intergenic distances per species and genomic region (log10 scaled) and **(B)** the results of pairwise statistical comparisons between all species and genomic regions. Distributions of **(C)** mean intron lengths per annotated genes for each species and genomic region (log10 scaled) and **(D)** the results of pairwise statistical comparisons between all species and genomic regions. Distributions of **(E)** per-gene GC3 for each species and genomic region and **(F)** the results of pairwise statistical comparisons between all species and genomic regions. Significant pairwise comparisons were based Dunn tests with Bonferroni-corrected *p*-values calculated across all three panels and all pairwise comparisons for repeat element composition (Supplementary Figure S10) are summarized above the diagonal (N = 5,349 comparisons; *p* ≤ 0.05; red cells). Differences in the pairwise median measures of genomic composition (i.e., effect sizes) are summarized below the diagonal for each pairwise comparison. The color scales to the right of each panel indicate the effect sizes with units based on the original metric (see *y*-axis titles on panels **A**,**C**, and **E**). See Supplementary Table S11 for the summary statistics underlying intergenic distance, intron length, and GC3 distributions and the results of pairwise statistical comparisons between each genomic region (MHC and non-MHC) and amniote species.

We also compared several attributes of genomic composition between MHC and non-MHC regions of the two anole species and the four other amniotes. We found significant differences between genomic regions and species in intergenic distances (Figure 7A; $\chi_{Kruskal-Wallis}^{2}\left(11\right)=11,878.7,\;p\leq 0.001$ ), mean per-gene intron lengths (Figure 7B; $\chi_{Kruskal-Wallis}^{2}\left(11\right)=9,997.7,\;p\leq 0.001$ ), and per-gene percent GC3 (Figure 7C; $\chi_{Kruskal-Wallis}^{2}\left(11\right)=6,711.2,\;p\leq 0.001$ ). As expected, in non-MHC regions, intergenic distances and intron lengths were smaller in the two bird genomes than in the genomes of mammals and non-avian reptiles. Moreover, differences in intergenic distances and intron lengths were noticeably larger in MHC regions vs non-MHC regions for all non-squamate species (mean percent decrease of 67% for both metrics), whereas the two anole species displayed more modest reductions of these metrics in MHC regions (mean percent decrease of 33.8% and 23.1%, respectively; Figures 7A,B). Per-gene percent GC3 was higher in MHC regions *versus* non-MHC regions across all species. However, characteristics of GC content were more similar in MHC and non-MHC regions in the two anole species and humans (mean percent decrease of 15.4%) than they were in the other species evaluated here (mean percent decrease of 28%; Figure 7C). Overall, squamate genomes more closely resemble mammalian and tuatara genomes in repeat content and GC3, helping to explain patterns of MHC composition largely shared between squamates and non-avian amniotes. However, greater similarities in the composition of MHC regions relative to non-MHC regions are evident in Squamata, suggesting that patterns of genomic evolution in the MHC region are also unique in this clade.

### Gene orthology across Amniota

The Ensembl database recorded 73 genes in the MHC region between the five species with 1-to-1 relationships and 39 genes with 1-to-many or many-to-many relationships (Supplementary Figure S11; Supplementary Table S12). 68 and 22 genes showed 1-to-1 orthology and 1-to-many or many-to-many between human and green anole or tuatara, respectively, whereas fewer (N = 28 1-to-1 orthologs and N = 27 non-1-to-1 orthologs) were shared between human and chicken or zebra finch. Only five genes showed 1-to-1 orthology across all five species: *GABBR1* (human ortholog Ensembl ID ENSG00000204681), *NELFE* (ENSG00000204356), *SKIV2L* (ENSG00000204351), *TUBB* (ENSG00000196230), and *VARS2* (ENSG00000137411). Four genes showed 1-to-many or many-to-many relationships across all species compared: *C4A* (ENSG00000244731), *C4B* (ENSG00000224389), *CFB* (ENSG00000243649), and *TAP2* (ENSG00000204267). The brown anole genome is not yet included in Ensembl so similar information was not available for this species. However, a reciprocal best-BLAST between the annotated proteins of the green and brown anole genomes identified 4 of these nine genes in the brown anole, and we also identified five more genes based on a stringent one-way BLAST between the anole protein annotations, indicating that these nine genes are also present in the brown anole genome. Given human was used as a reference for our search of homology, no genes were unique to any species except humans, but the green anole was the only species with genes that lacked orthology in any of the other three species (N = 9 1-to-1 orthologs and N = 8 non-1-to-1 orthologs; Supplementary Figure S11; Supplementary Table S12). Genes shared in only the human and green anole genomes could arise from incomplete assemblies or annotations or true patterns of reduced composition of genes in the MHC regions of the tuatara and bird genomes.

### Evolutionary histories of MHC homologs

**FIGURE 8:**
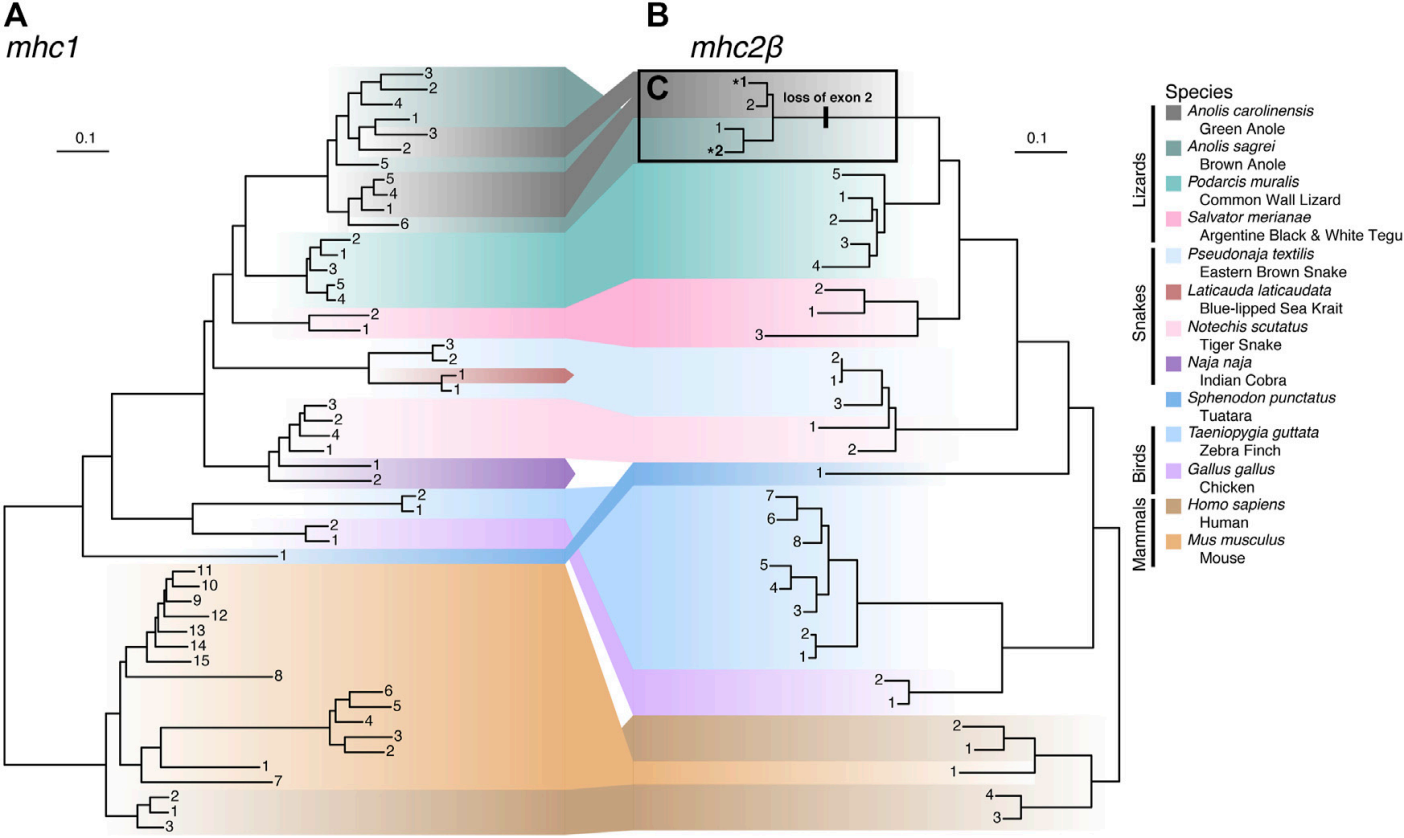
Phylogenies of **(A)** *mhc1* and **(B)** *mhc2β* homologs from 13 amniote species. Gene homologs are labeled with sequential numbers for each species. See Supplementary Table S13 for associations between sequential numbers and annotation gene IDs for each species and gene. **(C)** Anole homologs of *mhc2β* lacking exon 2 are indicated with bolding and an asterisk (*) and the branch where the loss of this exon putatively occurred is also indicated.

To illuminate genetic relationships among MHC homologs in anoles and more broadly across Squamata, we constructed phylogenetic trees of exons 2 and 3 for *mhc1* and *mhc2β* (Figure 8 and Supplementary Table S13). For *mhc1*, species homologs are generally clustered into monophyletic clades, though we observed paraphyly of homologs from the Indian cobra (*Naja naja*), eastern brown snake (*Pseudonaja textilis*), and brown anole (*A. sagrei*) and polyphyletic relationships between homologs from the green anole (*A. carolinensis*; Figure 8A), which are nested among homologs from the brown anole. Snake homologs from four Elapidae species (*Laticauda laticaudata*, *N. naja*, *Notechis scutatus*, and *P. textilis*) comprise two major lineages that form a paraphyletic group while homologs from the lizard species (*A. carolinensis*, *A. sagrei*, *Podarcis muralis*, and *Salvator merianae*) form a monophyletic clade (Figure 8A). Contrary to the known phylogenetic relationships between mammals, birds, tuatara, and squamates (see Figure 1A), *mhc1* homologs from birds and squamates are clustered phylogenetically. Species homologs of *mhc2β* are also generally clustered into monophyletic clades, though paraphyly is observed among homologs from tiger snake (*N. scutatus*) and human, with the lone mouse *mhc2β* homolog nested amongst homologs from human (Figure 8B). As opposed to *mhc1*, *mhc2β* homologs from the tuatara and squamates cluster together, resulting in a topology that agrees with the accepted phylogenetic relationships between these major amniote lineages (Figure 8B). Similar phylogenies were resolved from alignments where we excluded hypervariable or putatively selected sites, namely the green anole-specific peptide binding codons of *mhc1* and exon 2 of *mhc2β*, which indicates that these sites have little effect on the tree topologies.

**FIGURE 9:**
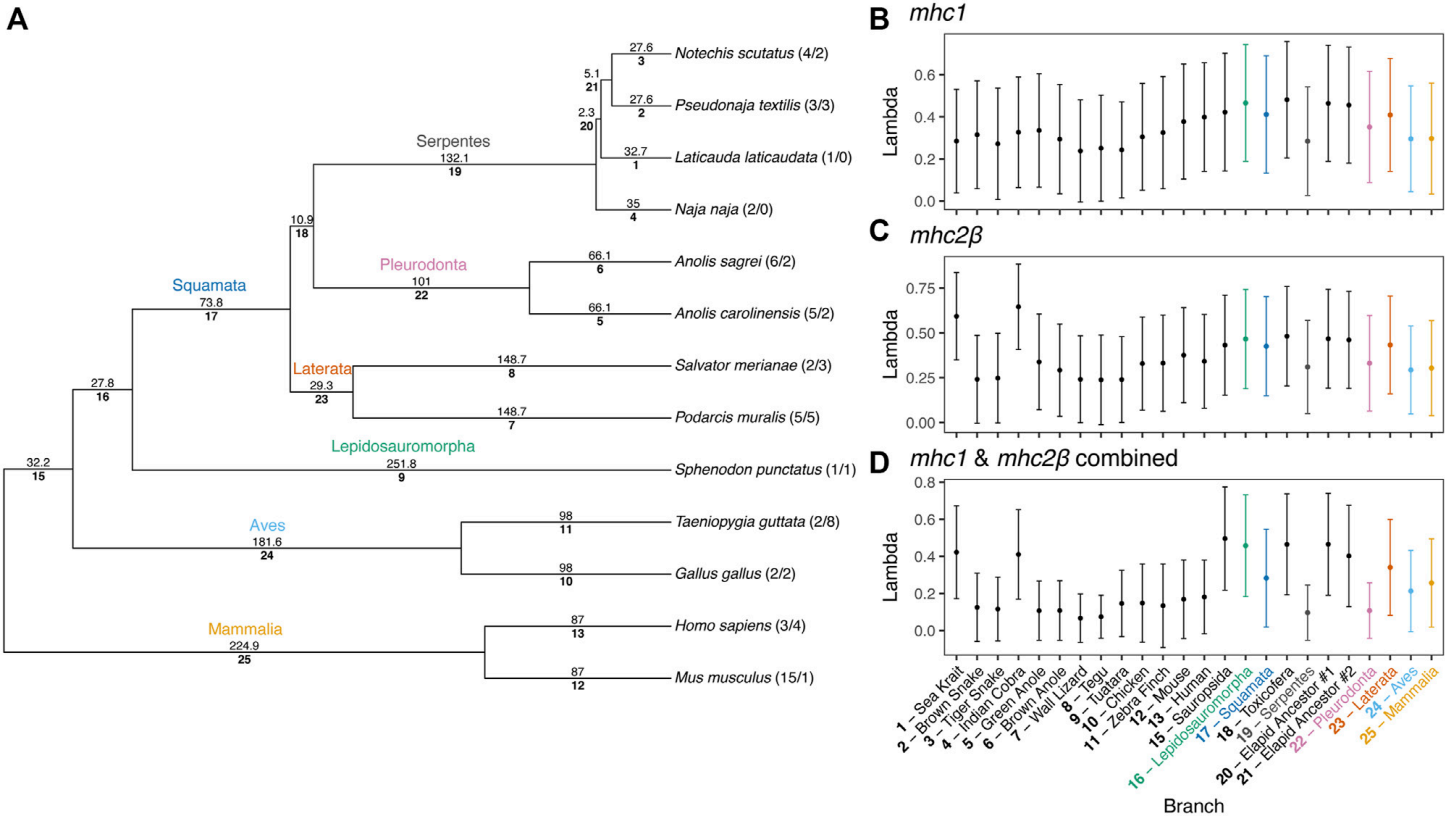
Results of a Bayesian analyses of gene family evolution of MHC gene families across 13 amniote species. **(A)** Guide phylogeny used to analyze gene family evolution. Numbers above each branch indicate time in millions of years, as determined from Timetree.org (Hedges et al., 2006; Kumar et al., 2017). Numbers below each branch indicate the index number of the branch used in parsing the Bayesian output. Numbers after the taxa names at the tips of the phylogeny indicate the number of *mhc1* and *mhc2β* gene copies per species, respectively. Branch indices are used to indicate the median (point) and standard deviation (error bars) of birth-death rates (lambda, λ) for each branch in the phylogeny for **(B)** *mhc1*, **(C)** *mhc2β*, and **(D)** a combined dataset of *mhc1* and *mhc2β*. Common names for each species are also supplied for terminal branches and clade names are provided for internal branches.

To estimate rates of MHC gene duplication and loss across amniotes and understand trends within squamates, we conducted a Bayesian analysis of gene family numbers across 13 species for *mhc1*, *mhc2β*, and the two families combined. We estimated lambda, the birth-death rate, assuming a model in which lambda can vary across branches of the amniote tree (Figure 9). Analyzing only one or two gene families resulted in large uncertainties in estimates of lambda across the tree. Nonetheless, for both *mhc1* and *mhc2β*, we estimated the highest rates on the deep branches leading to Sauropsida and Lepidosauromorpha, as well as the terminal branches leading to the sea krait (*L. laticaudata*) and Indian cobra (*N. naja*) for *mhc2β* and the combined data (Figure 9). The high estimated value for lambda in the snake lineages likely stems from our inability to find any full-length MHC class II genes in either of these taxa. A high lambda value suggests high turnover of these genes, in some cases leading to complete loss of the family. Although our inability to find any *mhc2β* genes in snakes may stem from poor assemblies, we suspect that the true numbers are quite low regardless, thus departing drastically from the pattern in passerine birds, which often possess scores of *mhc2β* genes (Minias et al., 2019).

### Anole-specific loss of exons in a subset *mhc2β* homologs

While building the multi-species alignment of *mhc2β* homologs, we initially identified only a single putative *mhc2β* homolog for the green anole in the Ensembl database lacking a sequence region homologous with exon 2 of this gene. In the brown anole, two putative *mhc2β* homologs were identified, with one being full length while the other also lacking exon 2, as we observed in the green anole. This led us to speculate that both anole species have two *mhc2β* homologs each and that one homolog from each species lacks exon 2. Several analyses rule out the possibility that genome assembly artifacts, such as gaps or incomplete genome annotations, caused gene models lacking exon 2 in each species (see Supplementary Material).

To investigate whether an incomplete genome assembly of the MHC region in the green anole has led to a pattern of a missing second, full length *mhc2β* homolog in the green anole, we gathered a cross-tissue gene expression dataset from the Bgee database (Bastian et al., 2021), filtered away reads that did not map to the full length *mhc2β* homolog of the brown anole (gene annotation ID ANOSAGT006753), and assembled these reads into transcripts (see Supplementary Material). We recovered several assembly contigs with homology to *mhc2β*, including one (Trinity assembly ID TRINITY_DN0_c0_g1_i5) that aligned well to our existing cross-species dataset across both exons 2 and 3. The presence of an assembled transcript with an intact exon 2 supports the hypothesis that a genome assembly artifact, namely a lack of an assembly for the region containing the second, full-length *mhc2β* homolog in the existing green anole genome assembly, accounts for the missing *mhc2β* homolog in the green anole. Therefore, it appears that each anole species genome contains a full-length *mhc2β* homolog and a second homolog lacking exon 2, which putatively evolved on the branch to *Anolis* and is a pattern unique among the other squamate species evaluated in this study (Figure 8C).

### Patterns of codon-specific natural selection in anoles

**FIGURE 10:**
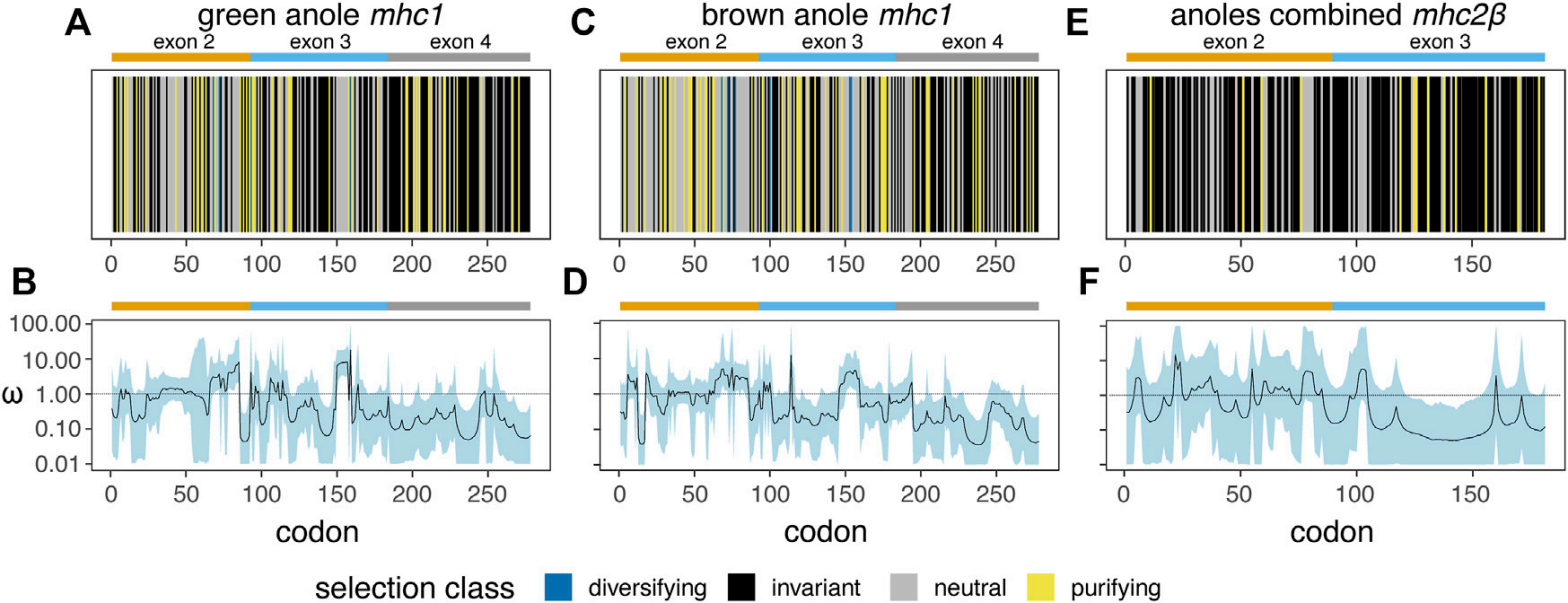
Results of two analysis of natural selection - HyPhy Fixed Effects Likelihood (FEL; **(A)**, **(C)**, and **(E)**) and omegaMap **(B)**, **(D)**, and **(F)** - for *mhc1* in the green anole (*A. carolinensis*; **(A and B)** and the brown anole (*A. sagrei*; **(C and D)** and for *mhc2β* in a combined dataset of both anole species **(E and F)**. For the FEL analysis **(A)**, **(C)**, and **(E)**, a vertical line represents each codon and is colored based on the inferred codon classification. The margins and span of exons 2 (orange), 3 (blue), and 4 (grey) are indicated with bars over each panel. See Supplementary Table S14 for the per-codon results of the FEL analysis of each dataset.

Based on separate analyses of anole *mhc1* homologs using a phylogenetic context (HyPhy FEL) or accounting for recombination (omegaMap), we detected signals of positive or diversifying selection in both anole species (Figure 10). In contrast, *mhc2β* showed no signal for positive or diversifying selection, although this pattern could be due in part to the low number of homologs present, even in a combined anole-species dataset (Figures 10A–C vs Figures 10D–F). A second FEL analysis of the combined-anole *mhc2β* dataset, where a *Podarcis* sequence was set as an outgroup, also yielded no codons with evidence of diversifying selection, although many codons that were invariant in the anole-only dataset were inferred to be evolving neutrally or under purifying selection in this expanded dataset (Figure 10C vs Supplementary Figure S12). Qualitatively, we observed correlations in the signals of selection detected in the FEL and omegaMap analyses for each species and alignment, with codons inferred to be under diversifying selection in the FEL analysis corresponding to areas of the alignment where the measure of omega (ω) from omegaMap increased. For *mhc1*, we also observed evidence of diversifying selection in similar regions of the two-exon amino acid alignment in both anole species, such as in the regions around codons 70 and 155 corresponding to exons 2 and 3, respectively (Figure 10; Supplementary Table S14).

Based on the FEL analysis of *mhc1*, a small number of codons were positively selected: four (two codons each from exons 2 and 3) and six (three codons each from exons 2 and 3) codons in the green anole and brown anole, respectively. Two of these codon positions, 68 and 72, were identified as under diversifying selection in both species based on the FEL analysis (Figure 10; Supplementary Table S14). We cross-referenced the results of our FEL analysis of each anole species with known peptide binding codons inferred from previous crystal structures of mhc1 proteins from green anole, chicken, and human (Supplementary Figures S13A-C, respectively; Supplementary Table S15), where many of the same codons are involved in peptide binding across all three species. Several of the codons interacting with peptides across two or more binding pockets of the mhc1 protein are under purifying or diversifying selection in one or both anole species: 1) codon 68 functions in the A and B pockets of the chicken and human proteins and is under diversifying selection in both anole species; 2) codon 72 functions in the B pocket of the green anole protein, the B and CDE pockets of the chicken protein, and the B and C pockets of the human protein and is under diversifying selection in both species; 3) codon 100 functions in the C and D pocket of the green anole protein, the A, B, and CDE pockets of the chicken protein, and the A, B, and D pockets of the human protein and is under purifying selection in the green anole and diversifying selection in the brown anole; and 4) codon 160 functions in the A and D pockets of the green anole protein, the A and CDE pockets of the chicken protein, and the A and D pockets of the human protein and is under purifying selection in the green anole only (Supplementary Figure S13; Supplementary Table S9).

## Discussion

Our results provide a first overview of the location and structure of the MHC regions in two anole species and squamates in general. Using two independent datasets and approaches, one based on BAC screening and sequencing and the other on BLAST-derived inference of homology, we localized the core MHC region to one end of chromosome 2 in both species; additional signals for homology were also evident on other chromosomes (Figures 2, 3 and Supplementary Figures S4-S7). We produced detailed gene maps for both anole species and several other amniote species (Figure 4), illuminating MHC composition in squamates and improving our understanding of the MHCs of other species. Although our gene map for the human MHC contained a subset of 103 genes (of the ∼260 annotated MHC genes), at 3.9 Mb, our estimate of size of the human was similar to previous estimates (3.5 Mb; The MHC sequencing consortium, 1999; Trowsdale and Knight, 2013). Our characterization of the chicken MHC differed more significantly from previous estimates (Shiina et al., 2007; Fulton et al., 2016), with a total gene count of 20 genes (45 genes were previously identified) and an MHC size of 640 kb, over twice as large as previous estimates of 242 kb (Shiina et al., 2007). Genome assembly quality impacted our ability to characterize MHC composition, leading to differences in our estimates of composition and size of MHCs for tuatara and zebra finch *versus* those from previous investigations (Balakrishnan et al., 2010; Miller et al., 2015; Gemmell et al., 2020), although our results improved on previous MHC maps for these two species by identifying greater numbers of MHC genes. Indeed, genome assembly quality impacted our ability to annotate anole MHCs as well, resulting in a higher-quality MHC annotation in the better-assembled brown anole genome, even with the green anole scaffolds we were able to place and orient in this study. Our analyses of k-mer coverage across the MHC regions of both anole species also indicated that assembly artifacts may still be present in these regions, and further genome sequencing of both species, especially using long read technologies, may help eliminate these artifacts. As increasing numbers of reference genomes are produced for non-traditional model species, care must be taken to control for the impacts of genome assembly quality when characterizing MHCs and, especially, when making cross-species comparisons.

As observed in many amniote species thus far, most previously identified MHC genes are found in a single core MHC region. However, MHC homology was observed on other macrochromosomes, especially chromosomes 1 and 4, and the putative ortholog for the gene *DMA*, an MHC class II gene that is normally found in the core MHC region in mammals and birds, has relocated to a different region on chromosome 4 in the anole genomes. Relocations of MHC genes to other genomic regions have been observed in other taxa, such as the relocation of class I genes away from the core wallaby MHC region (Deakin et al., 2007; Siddle et al., 2009). Class I genes have also been observed on two chromosomes in the tuatara (Miller et al., 2015), the sister lineage to all squamates, and the recent analysis of the tuatara reference genome found evidence for class I and II genes located outside the core MHC region in this species (Gemmell et al., 2020). These patterns suggest that class I or II gene translocations are an important aspect of MHC evolution among Lepidosauromorpha, although further investigations will be needed to better understand these patterns.

The presence or absence of genes in the MHC region of anole species that have also been identified in the MHC region of other amniotes may shed light on the evolution of the squamate MHC region or distinct characteristics of immunity evident in squamates. As part of our annotation of genes within the anole MHC region, we found several genes known from the mammalian MHC that are missing in the genomes of one or both anole species. Among the missing MHC genes, those genes missing in both anole species are of evolutionary interest and most likely to have been lost in squamates generally or *Anolis* specifically. Two related genes, *LTA* and *LTB*, which are also closely related to *TNF*, were missing in both anole species. *LTA* and *LTB* interact as part of their role in innate immunity, where they help prevent tumor growth (Fernandes et al., 2016). *C4B*, another gene unannotated in both anole species (but potentially present based on BLAST searches), interacts with the antigen-antibody complex and other complement components (Mortensen et al., 2015). Several genes with numerous functions were identified as missing in only one anole species. Both *C2* and *C4A*, which are functionally related to the *C4B* gene missing in both anole species, were missing in the green anole genome, a pattern suggesting that the complement system, a component of innate immunity, may differ in squamates. Several genes are also duplicated in the MHC region of one or both anole species compared to the human MHC region. Based on RefSeq annotations (O’Leary et al., 2016), genes duplicated in one or both anoles have a range of functions: *DDR1* and *TNXB* are involved in extra-cellular arrangement or communication of cells, *GTF2H4* is involved in RNA polymerase II activity, *RPP21* is a protein subunit of nuclear ribonuclease P, *AGPAT1* is an enzyme involved with signal transduction and lipid biosynthesis that converts lysophosphatidic acid into phosphatidic acid, and *VWA7* is a homolog for von Willebrand factor, which is involved in platelet adhesion. However, genome assemblies and annotations for both anole genomes are still incomplete and likely to change with additional data, and potential assembly artifacts in the MHC region, such as retained heterozygosity or the collapse of highly similar contigs/scaffolds, may contribute to patterns of missing or duplicated genes. More reference genomes are being sequenced for squamate reptiles, so additional work is necessary to definitively determine whether these genes are missing or duplicated in these anole species or squamates more generally.

Orthology, especially 1-to-1 orthology, between human MHC genes and annotated genes in four other species is only confidently ascertained in a small portion of genes in the extended MHC across all species. These patterns are influenced by the small number of MHC genes in the chicken, but zebra finch also appears to have a relatively streamlined MHC with fewer genes than is commonly observed in mammals, which explains the smaller number of overlaps between zebra finch and other species. Indeed, the highest number of inferred orthologous relationships was between human, green anole, and tuatara, although a significant number of human genes in the extended MHC show no orthology in any other species (Supplementary Figure S11). A largely intact and syntenic MHC region in squamates also supports the hypothesis that the ancestral MHC region in amniotes contained a relatively full complement of MHC genes that has been retained largely intact in most extant amniote clades, a hypothesis that is supported by the largely complete complex of extended MHC genes known from amphibians such as the frog *Xenopus laevis* (Ohta et al., 2006). In total, our findings underscore the unique nature of the streamlined avian MHC, especially in the case of the minimal essential MHC in chicken (Kaufman et al., 1999; Kaufman, 2018), and provide important context on MHC composition and structure for the approximately 40% of known amniotes classified as squamates.

We found that structural and compositional features of squamate MHC regions were similar to mammals and the tuatara, but still distinct in certain ways. GC content in the MHC regions of anoles was approximately 40% (Figure 5) and GC3 of all protein-coding genes in the MHC region of the green anole was approximately 50% or greater (Figure 7). Although GC3 is significantly inflated in the MHC relative to genome-wide patterns in all species, the difference between GC3 in MHC regions and genome-wide is relatively low in squamates (Figure 7). Additional comparative analyses will be needed to determine whether patterns of GC content in MHC and non-MHC regions are unique in Squamata, although previous studies have found that squamates have unique signatures of GC content relative to other vertebrates (Janes et al., 2010; Alföldi et al., 2011; Fujita et al., 2011; Castoe et al., 2013; Figuet et al., 2015). Although repeat elements in the MHC have previously been quantified in individual species (e.g., Matsuo and Nonaka, 2004; Moon et al., 2005; Wan et al., 2009; Chen et al., 2015), only a single study has conducted an analysis of MHC region repetitive content across multiple species (i.e., mammals; Yuhki et al., 2003) and our study is the first to encompass species from across Amniota. As in mammals and tuatara, anole MHC regions, and genomes in general, contain appreciable proportions of LINE retrotransposons (Shedlock, 2006; Alföldi et al., 2011; Pasquesi et al., 2018; Figure 6 and Supplementary Figure S10). However, squamate genomes and MHC regions have relatively high proportions of DNA transposons, which are uncommon in mammals, and low densities of SINE retrotransposons that are in high copy-number in mammals. Tuatara shares these same patterns, although DNA transposons, which are similarly abundant genome-wide as they are in squamates, are lacking in the tuatara MHC region.

For DNA transposons and LINE elements, the two most common repeats in squamate genomes, abundances tended to be relatively higher in the MHC region *versus* genome-wide in squamate genomes, while this pattern was reversed in the MHC regions of mammals and birds. The explanation for such a pattern is currently unknown but it could be driven by both adaptive and non-adaptive processes. Repeat elements are known to seed regulatory sequences throughout the genome (Feschotte, 2008), and the buildup of repeat elements in the MHC region could be selectively advantageous for controlling the expression of nearby genes. Instead, repeat insertion in the MHC region could largely be a product of neutral evolution where abundances are driven by insertion and deletion dynamics in the genome. Rates of recombination could drive differences in the composition of repeats in the MHC and genome-wide. In human, rates of recombination are much lower in the MHC *versus* the rest of the genome (de Bakker et al., 2006). The location of the anole MHC at the end of a chromosome and the well-documented pattern of increased recombination rates towards the telomeric regions of chromosomes (Haenel et al., 2018) suggests that recombination rates in the MHC region may be more similar, or perhaps higher, than the genome-wide average. Indeed, a population genomic investigation of the green anole inferred relatively higher rates of recombination at end of each chromosome (Bourgeois et al., 2019). Moreover, Bourgeois et al. (2020) used population genomic data from the green anole to detect a positive correlation between recombination rate and repeat frequency. Together, these previous conclusions and our results strongly suggest that the distal chromosomal location of the anole MHC region may result in fundamental differences in the rates of recombination in the MHCs of squamates and other amniotes that manifest as distinct MHC repeat landscapes. Patterns of repeat element abundance therefore suggest that the MHC regions of squamates are evolving in unique ways, which may contribute to the relatively large intergenic distances and long intron lengths observed in squamates (Figure 7). These large intergenic distances and long intron lengths, in turn, may explain the significantly larger footprint of the squamate MHC, which was several times larger than any previously evaluated species (Figure 4; Supplementary Table S9). Given the entire core MHC region is assembled on a single scaffold in the brown anole, it is unlikely that our estimates of the size of the extended MHC region are erroneously high. Further studies are needed to determine whether these patterns of MHC composition and structure generalize across squamates or are observed in other amniote lineages that have not yet been studied.

Our analysis of the evolutionary history of *mhc1* and *mhc2β* homologs from the two anole genomes and the genomes of six other squamates, tuatara, two birds, and two mammals yielded phylogenies in which species homologs generally formed monophyletic groups (Figure 8). The exceptions to this pattern were evident in lineages with higher taxonomic density, such as the lineage including two relatively closely related anole species, the lineages encompassing up to four species of snakes from the family Elapidae, and the lineage including human and mouse. This topological pattern suggests that gene duplications have likely occurred after divergence between the major amniote lineages and often before subfamily divergence in the anole, elapid, and mammalian lineages (Nei et al., 1997). Given our analysis includes a small number of representatives from major amniote lineages, this result is expected given the proto-amniote ancestor of all taxa likely possessed a single *mhc1* and *mhc2β* gene copy (Ohta et al., 2000, 2006; Flajnik and Kasahara, 2001). The topology of both MHC genes is similar to the known topology of these major amniote lineages, with the exception of the sister relationship between bird and squamate homologs in *mhc1* (the accepted amniote phylogeny resolves the tuatara as the sister lineage of Squamata, as was derived from the *mhc2β* dataset; Figure 8). Copy number of species-specific homologs varied between *mhc1* and *mhc2β*. Moreover, for two elapid snakes (*Laticauda laticaudata* and *Naja naja*), we were only able to recover homologs for *mhc1*, which is likely due to an incomplete genome assembly or annotation rather than actual loss of these genes in these species. Apparently true losses of whole classes of MHC genes have been documented in some vertebrates (Star et al., 2011), and confirmed and extended through low coverage sequencing, such as the loss of MHC class II genes in gadiform fish (Malmstrøm et al., 2016). The paucity or absence of *mhc2β* genes in snakes led to estimates of a relatively high rate of turnover (lambda, λ) for Bayesian gene family evolution in these taxa (Figure 9; Liu et al., 2011). Despite the poor assembly quality in some of our analyzed taxa, *mhc1* genes generally have higher copy number in squamates and mammals while *mhc2β* have higher copy number in birds. We suspect that assembly or annotation artifacts may lead to biased counts of species homologs contributing to these trends, but the overall contrasts between taxa are likely robust (Figure 8; Minias et al., 2019).

Our investigation of the squamate MHC also uncovered strong evidence for the loss of exon 2 in one of two *mhc2β* homologs of both anole species (Figure 8C), a pattern that does not appear to be a result of an inaccurate gene annotation or a poor genome assembly in the region of the genome containing these genes. Our data indicate that this evolutionary pattern is unique to the broad lineage including anoles, but further investigations of genomes of anoles and other species will be needed to determine the phylogenetic extent of this feature within Iguania. However, given that the green and brown anole are relatively distantly related among anoles (divergence of ∼39 million years; see also Poe et al. (2017)), it is likely this pattern encompasses at least the genus *Anolis*. Given that exon 2 forms a significant portion of the *mhc2β* peptide binding domain (Brown et al., 1988; Madden, 1995), this loss likely has large functional consequences that will require future studies to uncover. It is not uncommon to observe deletions of coding regions or premature stop codons across distinct copies or alleles of MHC genes due, often, to pseudogenization (Vanin, 1985; Hess et al., 2000; Sawai et al., 2008) and previous studies have revealed gene expression isoforms lacking certain exons due to alternative splicing (Ishitani and Geraghty, 1992; Guidry and Stroynowski, 2005; Belicha-Villanueva et al., 2010; Dai et al., 2014; Voorter et al., 2016).

Our analysis of selection in *mhc1* homologs recovered evidence for both purifying and diversifying selection across many codons (Figure 10 and Supplementary Figure S13). For *mhc2β*, on the other hand, we only recovered evidence of purifying selection acting on some codons, which is likely due to the small sample size of our dataset for this gene (N = 1 full-length homolog from each anole species; Figures 10E, F). We find a proportion of selected *mhc1* codons fall in positions that are known to comprise the peptide binding pockets of the green anole, chicken, or human (Supplementary Figures S13A-C, respectively), which supports the conclusion that lineage-specific selection is operating in anoles that may impact the recognition of pathogenic peptides. A large proportion of the codons contributing to two or more peptide binding pockets show evidence of either purifying or diversifying selection in one or both anole species. Such a pattern indicates that broadly important codons functioning in peptide binding across multiple pockets may be subject to selective pressures in anoles and, perhaps, other species. Interestingly, across the known peptide binding codons derived from crystal structures of *mhc1* in human, chicken and green anole that were profiled in the green and brown anole, we observed evidence for diversifying selection impacting codons in pockets A-E. However, no evidence of diversifying selection was evident in codons contributing to pocket F, where codons are generally invariable or are evolving neutrally, with the single exception of purifying selection impacting codon 118 in both anole species (Supplementary Figure S13). The lack of diversifying selection across codons that contribute to pocket F in one or more amniote species suggests that this pocket may be subject to more evolutionary constraint in the two anole species included in this analysis and perhaps more broadly across anole or other amniotes. In line with this hypothesis, pocket F usually binds the C-terminus peptide residue (Madden, 1995) and is seemingly relatively conserved due to its role as an anchor pocket across vertebrate species studied to date (Koch et al., 2007; Wang et al., 2021).

Overall, this study fills a significant gap in our understanding of amniote immunity by thoroughly characterizing for the first time the structure and composition of the MHC in Squamata. To date, studies of squamate immunity generally, and MHC specifically, have lagged significantly behind similar areas of research focused on other amniotes (Figure 1). Over 10 years passed between the original publication of the first squamate genome, that of the green anole, and this detailed study of MHC characteristics in a squamate species. In contrast, the structure and composition of the MHCs in other vertebrates were well described alongside or soon after the publication of early reference genomes in these lineages (e.g., Kaufman et al., 1999; The MHC sequencing consortium, 1999; Ohta et al., 2006) and in some cases (e.g., Steinmetz et al., 1982; Weiss et al., 1984), substantial knowledge was derived even before modern genome sequencing. Significant progress has even been made characterizing the MHC and immune biology in much smaller amniote clades like crocodilians, turtles, and tuatara. This research bias restricts our knowledge of the squamate MHC and immunity, though some early studies indicate that squamates may have unique immunological characteristics, such as a lack of γδ T cells (Morrissey et al., 2022). Scientists’ ability to understand and respond to squamate-specific pathogens, such as the emerging snake fungal disease (Lorch et al., 2016; Franklinos et al., 2017), may also be hindered by limited knowledge of squamate MHC and immunity. Finally, scientists have been unable to harness the unique aspects of squamate natural history that could be linked to immunity or MHC biology both in focused studies of Squamata and comparative studies with other taxa. For instance, a more detailed understanding of the impact of endothermy on MHC biology may be possible through studying MHC in non-avian reptiles (Flajnik, 1996, 2018), the only ectothermic amniotes, of which squamates form a significant fraction of diversity. Greater understanding of the important role that MHC plays in pregnancy, which has been thoroughly studied in mammals (Bainbridge, 2000; Moffett and Loke, 2006), may also be possible by studying the repeated evolution of viviparity in Squamata (Shine, 1983; Blackburn, 2000; Stewart and Thompson, 2000; Thompson and Speake, 2006; Sites et al., 2011). Our work, therefore, builds an important foundation upon which additional studies can be conducted on the MHC in squamates and more broadly.

## Data Availability

The datasets presented in this study can be found in online repositories. Assembled BAC contigs have been deposited in GenBank, accession numbers OM796078–OM796082. Large genomics datasets underlying analyses have been deposited in a Figshare repository, DOI https://doi.org/10.6084/m9.figshare.21200119.
