# Supplementary material for: Structure and evolution of the squamate major histocompatibility complex as revealed by two Anolis lizard genomes
Source: Front Genet. 2022 Nov 8;13:979746. doi: 10.3389/fgene.2022.979746 (PMC9679377; doi:10.3389/fgene.2022.979746)
Supplement: Supplementary file 1 [file DataSheet1.docx]

# **Supplementary Materials: Structure and evolution of the squamate major histocompatibility complex as revealed by two *Anolis* lizard genomes**

Daren C. Card^1,2,*^, Andrew G. Van Camp^1,2^, Trenten Santonastaso^3^, Michael I. Jensen-Seaman^4^, Nicola M. Anthony^3^, and Scott V. Edwards^1,2,*^

^1^ Department of Organismic & Evolutionary Biology, Harvard University, Cambridge, MA, USA

^2^ Museum of Comparative Zoology, Harvard University, Cambridge, MA, USA

^3^ Department of Biological Sciences, University of New Orleans, New Orleans, LA, USA

^4^ Department of Biological Sciences, Duquesne University, Pittsburgh, PA, USA

^*^ Correspondance:

Daren C. Card: dcard@fas.harvard.edu

## **Supplementary Materials and Methods**

### **Quantification of literature on amniote MHC**

We performed a literature search to quantify the amount of MHC research in each major amniote clades. Our literature search was completed on March 10, 2022. Using both Google Scholar and Web of Science, we used the query ("MHC" OR "major histocompatibility complex") AND {clade taxonomic terms} where {clade taxonomic terms} varied for each lineage as follows:

Squamata = (“Squamata” OR “squamate” OR “lizard” OR “snake”)

Mammalia = (“Mammalia” OR “mammal” OR “human” OR “mouse”)

Crocodylia = (“Crocodilia” OR “Crocodylia” OR “crocodilian” OR “crocodile”)

Sphenodontia = (“Rhynchocephalia” OR “Sphenodontia” OR “tuatara”)

Testudines = (“Testudinata” OR “Testudines” OR “Chelonia” OR “turtle”)

Aves = (“birds” OR “Aves” OR “chicken”)

### **Assessing genome assembly quality**

We gathered data on green and brown anole genome contiguity (scaffold N50s) and completeness (presence of 3,354 vertebrate BUSCOs from vertebrata_odb10; Manni et al., 2021) from Geneva et al. (2021). However, broad measures of genome assembly contiguity and completeness may overlook assembly artifacts like retained haplotypes in complex genomic regions like the MHC. To evaluate assembly quality of the MHC regions, we estimated the genome-wide coverage of 27-mers in the MHC regions of each genome using the SECT tool from KAT (Mapleson et al., 2017) with the options “-E -F”. We cross referenced each 27-mer with the positions of annotated repetitive content to classify high-depth k-mers as repetitive.

### **Selection of MHC loci for BAC screening and homology search in the green anole**

Query sequences for candidate class I and II loci within the MHC were retrieved from 66 species available in GenBank. We then identified homologous sequences in the *A. carolinensis* genome by first using the BLASTN algorithm (Altschul et al., 1990) and then using the BLAST-like alignment tool search (BLAT; Kent, 2002) to search any significant hits against UCSC Genome Browser version of AnoCar2.0 to determine the location and identity of these loci using the ENSEMBL (Howe et al., 2021), AUGUSTUS (Stanke et al., 2004), and GENSCAN (Burge and Karlin, 1997) data tracks. Positive hits were identified as having an e-value ≤ 10^-15^. From these initial surveys of the genome, we selected the following MHC immunity genes against which to design our overgo probes: MHC class I and class II (*mhc1*, *mhc2*); Tapasin (*tap2*)—antigen processing genes for *mhc2* presentation (Janeway Jr. et al., 2001); tenascin XB (*tnxb*)—mediates communication between the cell and the intracellular matrix and is located in the MHC class III region (Bristow et al., 1993); latent membrane protein (*lmp2*, *lmp7*)—MHC related genes which modify proteins for antigen presentation (Gaczynska et al., 1994); butyrophilin (*btn3*)—codes for a membrane protein with an extracellular immunoglobulin domain located in the extended MHC region (Rhodes et al., 2001); retinoid X receptor beta (*rxrb*)—mediates the effects of retinoic acid and is found in the MHC class II region (Fitzgibbon et al., 1993); benign lymphoepithelial cysts (*blec1*)—plays a part in immune function during HIV infection (Mourad et al., 1997); ubiquitin-protein ligase (*ring1*)—MHC class II related gene involved in blocking transcription of other genes (Bárdos et al., 2000); flotillin-1 (*flot1*)—associated with the reduction in tumors (Bickel et al., 1997; Zhang et al., 2013).

### **BAC library screening, sequencing, and assembly in *Anolis carolinensis***

Overlapping oligo (“overgo”) probes were designed to map to exons within candidate MHC loci using the following criteria: an eight base-pair (bp) overlap between forward and reverse oligos (Integrated DNA Technologies), ~50% GC content, a total of 40 bp overgo length, and a melting temperature of 68°C with no palindromic regions (see Supplementary Table 2 for the list of overgo probes). Each probe sequence was verified through a megaBLAST search of highly similar sequences in GenBank. Prior to screening, the oligos were diluted to a concentration of 20 µM. 5 µL of each forward and reverse oligo were combined and annealed under the following conditions: 80°C for 10 min., 37°C for 10 min., and 4°C until use. Following this step, 2.0 µL of each annealed oligo probe pair was mixed with 1.0 µL BSA (1 mg/mL), 2.0 µL Oligo Labeling Buffer (OLB), 0.5 µL [α-^32^P]dATP, 0.5 µL [α-^32^P]dCTP, 0.5 µL Klenow fragment, and 4.5 µL water in a total reaction volume of 11 µL which was incubated at room temperature for one hour. The reaction was stopped with the addition of 30 µL of Tris-EDTA (TE) buffer. Samples were run through Sephadex G-50 packed Nick Columns (GE Healthcare) to remove unincorporated nucleotides. A *Caenorhabditis elegans* overgo probe was used as a positive control and to orient the autoradiograph after development.

We screened nine high density membranes from the *A. carolinensis* genomic BAC library, representing approximately 9x genome coverage. Adhered to each membrane were 36,864 colonies representing 18,432 unique BAC clones, each spotted in duplicate. Inserts from this library were, on average, 140kb in length where a single membrane represents approximately 1x coverage of the *A. carolinensis* genome. To increase laboratory efficiency, we screened each probe against half of the library, resulting in each probe querying 4-5x coverage. Library membranes were first primed in 1x SSC at RT for 15 min, then placed in 200 mL hybridization bottles with 35 mL of Church’s Hyb solution (1% BSA, 1.0 mM EDTA, 0.4 M NaPO4 pH 7.2, 7% SDS) and incubated at 60.7°C for 30 min. Plastic mesh (“Flow Mesh”, Diversified Biotech) was interlaced between membranes to allow the hybridization solution to contact all surfaces of the membranes. Probes were denatured at 90°C for five minutes and diluted in 5 mL of Hyb solution before being added to the bottles containing library membranes. Membranes were incubated overnight at 60.7°C while rotating at 15 revolutions per minute. After incubation, membranes were washed three times in RT 1x SSC and incubated for 30 mins at 60.7°C in a final 1x SSC wash. This rinse procedure was repeated until radiation levels reached approximately 0.03 milliroentgens per hour (mR/hr) which provides a radiation strength adequate to expose but weak enough to not overexpose the autoradiograph film. The hybridized membranes were sealed in plastic wrap and exposed to autoradiographic film in an X-ray cassette for 24 – 72 hours at -80°C. Autoradiograph film was developed according to standard darkroom technique using Kodak™ fixer and developer (Rochester, NY).

Five BACs were selected for 454 sequencing using the following criteria: BAC clone 73F21 hybridized with *mhc2* loci; its SP6 BES mapped to scaffold Un_AAWZ02036018, which appears to contain an MHC II homolog, while the T7 BES comprises entirely transposable elements and is therefore unmappable. Based on the location and orientation of the SP6 BES and the size of Un_AAWZ02036018, we predicted BAC 73F21 to contain >100kb of sequence immediately flanking an MHC II locus. Clone 107B15 hybridized with both *mhc1* and *mhc2* loci; its SP6 BES mapped to the Zn-finger-rich chrUn_GL343809 of the AnoCar2.0 assembly less than 10kb from the end of this scaffold, suggesting that the remainder of the sequence of 107B15 is incomplete or poorly assembled in AnoCar2.0. Clone 118B8 had positive hybridizations with *mhc1*, *lmp7*, and *tap2* genes; the SP6 and T7 BESs for this BAC aligned to two different unmapped scaffolds (Un_344036 and Un_344838), both of which contained large gaps. Clone 134P17 hybridized with *mhc2* probes multiple times; its BESs mapped to a single contig in the AnoCar2.0 assembly (Un_GL343520), suggesting that this BAC is contained entirely within the contig. However, no MHC homologs appear to map to this contig which contains several large gaps, indicating that there may be *mhc2* loci within these gaps. Finally, clone 4115L2 hybridized with *mhc2* loci; its BESs mapped to two different unmapped scaffolds in AnoCar2.0 (Un_GL343775 and Un_GL343676).

BAC clones chosen for sequencing were obtained from BACPAC Resources as *E. coli* agar stabs. Cells were first grown in 2 mL of LB broth with 12.5 µg/µL chloramphenicol in a 10 mL culture tube shaking at 220 rpm at 37°C overnight. The next day, 150 µL was used to inoculate a 500 mL culture, again in LB with 12.5 µg/µL chloramphenicol, and incubated overnight at 220 rpm and 37°C. The cells were harvested by pelleting the cultures at 6000 g for 15 minutes at 4°C. BAC DNA was purified using the NucleoBond Xtra BAC kit (Clontech, CA), following manufacturer's instructions. To confirm the identity of each BAC DNA before sequencing, we used PCR to amplify a ~400bp region using primers designed from the BES under the following conditions: 2 µL 10x buffer, 0.5 µM of each dNTP, 2.5 µM MgCl_2_, 0.5 µM each primer, 0.5 U Taq and ~ 5 ng DNA in a 20 µL reaction, and under the following thermocycler regime: initial denaturation for 96°C for 2 minutes, then 35 cycles of: 94°C for 10 seconds, 56°C for 15 seconds, and 72°C for 30 seconds, with a final extension cycle of 72°C for 10 minutes. The PCR amplicons were Sanger sequenced with BigDye 3.1 (ThermoFisher Scientific, NY, USA), and run on an Applied Biosystems 3100 genetic analyzer.

BACs were sequenced at the Genomics and Proteomics Core Laboratories at the University of Pittsburgh at 60X coverage using a 454 FLX sequencer (Roche Diagnostics, IN). The resulting BAC sequences were demultiplexed using FASTX_BARCODE_SPLITTER v.0.0.13.2 and barcodes were removed using FASTX_TRIMMER (Gordon and Hannon, 2010). *E. coli* contamination was removed from the reads using SEQCLEAN (http://sourceforge.net/projects/seqclean/files/). Reads were assembled into contigs with RUNASSEMBLY (<http://454.com/products/analysis-software/index.asp>).

To determine the location of BACs, and thus MHC homology, in the *A. carolinensis* genome, we retrieved all BES sequences produced as part of the *A. carolinensis* genome sequencing project from the NCBI Trace Archive, along with the assembly data of these sequences from the Broad Institute’s FTP site. Of the 385,917 BESs available, 152,686 were mapped to a unique location in the AnoCar2.0 assembly (Alföldi et al., 2011). We mapped additional 76,988 BESs to the assembly with local BLAST by taking the single best hit with an *e-value* less than 10^-20^ and using the *windowmasker* option. The location of these 229,504 BESs, along with 81,646 BES pairs where BESs were linked if the forward and reverse sequences from a BAC were mapped to the same chromosome or contig within 250 kb of each other, and facing each other in the correct orientation can be visualized by adding the URL<https://raw.githubusercontent.com/seamanlab/Anolis/master/BES_BES_pairs.bed> as a custom track in the UCSC Genome Browser AnoCar2.0 assembly.

We also aligned contigs from the five sequenced BACs to the AnoCar2.0 assembly using BLAT. To avoid spurious alignments, repetitive elements in the contigs were masked with RepeatMasker v.4.0.6 with Repbase library 20150807 (Smit et al., 2013). Results from each BLAT alignment were ranked from highest to lowest alignment score and plotted according to rank. Alignments with high scores that appeared before the point of inflection (i.e., when alignment scores plateaued at lower values), which had noticeably better alignment scores, were accepted as high-quality, unique alignments to distinct regions in the genome assembly and were carried forward for further analysis. Those alignments after the point of inflection were treated as spurious and were removed from further analysis. Example graphs for an accepted and a rejected contig based on this criterion are provided for illustration (Supplementary Figure 1). Of those alignments that remained, we used the alignments with the highest BLAT alignment score for evaluating the location of the contig in the genomic assembly.

### **Gathering and assessing evidence for the placement and orientation of MHC region scaffolds in the green anole**

We used whole genome alignments between the green and brown anole genomes to guide the placement and orientation of scaffolds in the green anole MHC region. For each genome, we first produced a *de novo* library of repeat consensus sequences using RepeatModeler v. 2.0.2 (Flynn et al., 2020) with default settings. We soft-masked repeats in each genome using RepeatMasker v. 4.1.2-p1 (Smit et al., 2013) first using vertebrate repeat consensus sequences from Repbase v. 3.3 (2020-11-09; Jurka et al., 2005; Bao et al., 2015) and then using the appropriate species-specific *de novo* repeat library. The soft-masked genomes were aligned using Cactus v. 2.0.1 (Armstrong et al., 2020) with default settings and a two-species phylogeny with a divergence of 0.0955937, as inferred from the genome assemblies using Mash v. 2.2.1 (Ondov et al., 2016) with default settings. We manually assessed the resulting alignments for evidence of synteny between the two genomes that allows the placement of green anole scaffolds based on the increased contiguity of the brown anole genome. Where possible, we used these results to place and orient unplaced scaffolds in relation to the core MHC region identified on chromosome 2.

We also leveraged the long-range spatial information available from RNA-seq data to understand whether there was further support for the placement of scaffolds. To gather representative RNA-seq data for *A. carolinensis*, we queried the Bgee database and collected 47 NCBI SRA sequencing runs derived from 15 distinct tissues (Bastian et al., 2021). Raw RNA-seq reads were aligned to the green anole genome from NCBI using BWA v. 0.7.17-r1188 (Li and Durbin, 2009) with the MEM algorithm and default options. These mapping data, along the genome assembly and gene annotations from NCBI, were used to further scaffold the green anole assembly using AGOUTI v. 0.3.3-25-ga7e65d6 (Zhang et al., 2016) with default settings except for minMQ=20 and maxFracMM=0.05. We cross-referenced the resulting AGOUTI scaffolds with scaffolds that had homology with MHC genes based on our BLAST search.

Finally, we evaluated whether our BAC sequencing data spanned breakpoints between scaffolds in the existing green anole genome assembly. To aid in manually identifying co-linear blocks of homology, we annotated genes *ab initio* using MAKER 2.31.8 (Holt and Yandell, 2011) trained with mRNA and protein databases (NCBI, collected Jan 25, 2017) of teleosts, *X. tropicalis, A. carolinensis*, *T. guttata*, and *G. gallus*. We confirmed our predictions by aligning our contigs to the *A. carolinensis* assembly in the Genome Browser (UCSC) and NCBI, and noting published annotations (AUGUSTUS, ENSEMBL, GENESCAN) of those regions. We accepted an annotation if it was detected in *A. carolinensis* by at least two methods, or if it was detected in *A. carolinensis* and at least one other taxon. BLAST-aligned exons of annotated regions were manually curated into presumptive mRNA sequences and their amino acid translations. Gene naming conventions as outlined in Kusumi et al. (2011) were followed.

We assessed the evidence for placing and orienting scaffolds with MHC homology by gathering BES pairs that mapped to separate scaffolds in the green anole genome where at least one BES has mapped to a scaffold with significant evidence for MHC homology. For each set of BES pairs, we gathered corresponding BAC contigs, mapped them against the genome using BLASTn v. 2.2.28 (Altschul et al., 1990), with default settings, and assessed the identity, orientation, and ordering of each full BAC contig and the corresponding gene annotations. When we observed strong evidence for co-linearity of contigs and annotated genes across two or more green anole scaffold with MHC homology, we interpreted this pattern as strong support for synteny between these genomic scaffolds and manually arranged and oriented the scaffolds based on the evidence.

### **Manual identification of MHC genes**

For known MHC genes for human, mouse, and chicken from the literature (The MHC sequencing consortium, 1999; Kelley et al., 2005), we searched each gene ID against the Ensembl database (Howe et al., 2021; release 105) for the green anole, human, chicken, zebra finch, and tuatara genomes. We cross-referenced the location of identified gene models with those locations that showed significant homology with MHC genes based on our BLAST-based localization analysis to reduce the probability of spurious information for gene identities or orthology in the Ensembl database. For many MHC genes, the gene could be identified using direct gene ID searches against the relevant reference genome dataset, but for other genes, it was necessary to identify the target gene in human or chicken and use Ensembl-inferred orthology to detect the most likely gene model in zebra finch, tuatara, or green anole. We also manually scanned all gene models in regions with large numbers of MHC genes to identify additional MHC genes that were not located based on direct gene ID queries or inference of cross-species orthology. We cross-referenced the location of identified gene models with those locations that showed significant homology with MHC genes based on our BLAST-based localization analysis to reduce the probability of spurious information for gene identities or orthology in the Ensembl database. The brown anole reference genome is not yet included in Ensembl, so we manually assessed gene identifiers in the annotation GTF file for the genomic regions with large amounts of MHC homology. Gene identifiers were assigned by using translated protein sequences of gene models as a BLASTP query against the NCBI Vertebrata RefSeq protein database. We used db2db in bioDBnet (biological DataBase network) to translate RefSeq protein accession IDs to gene symbols. Once we had produced a complete gene map of MHC genes for each species, we estimated the size of the MHC as the distance between the first and last annotated MHC gene. When the MHC region was located on multiple scaffolds, we used the same procedure to estimate the size of the MHC per scaffold and summed across scaffolds to produce an estimate of the total MHC size.

We also attempted to estimate the total number of core *mhc1* and *mhc2* genes identified and published in the green and brown anole genome assemblies. To do so, we started with known *mhc1* and *mhc2* genes in

human (*HLA-A*, *HLA-B*, *HLA-C*, *HLA-DRA*, *HLA-DRB1*, *HLA-DRB5*, *HLA-DQA1*, *HLA-DQA2*, *HLA-DQB1*, *HLA-DQB2*, *HLA-DPA1*, and *HLA-DPB1*);

mouse (*H2-K1*, *H2-K2*, *H2-D1*, *H2-L*, *H2-Aa*, *H2-Ab1*, *H2-Ea*, *H2-Oa*, *H2-Ob*, *H2-Pa*, *H2-Pb*, *H2-Dma*, *H2-DMb1*, *H2-DMb2*, *H2-M2*, *H2-M3*, *H2-M5*, *H2-M6-ps*, *H2-M9*, H*2-M10.1*, *H2-M10.2*, *H2-M10.3*, *H2-M10.4*, *H2-M10.5*, *H2-M10.5-ps1*, *H2-M10.6*, *H2-Q1*, *H2-Q2*, *H2-Q3*, *H2-Q4*, *H2-Q5*, *H2-Q6*, *H2-Q7*, and *H2-Q10*); and

chicken (*DMA*, *DMB1*, *DMB2*, *BLB1*, *BLB2*, *BF1*, and *BF2*).

For each gene, we manually identified orthologous gene models for green anole, tuatara, and zebra finch using Ensembl release 105. We also searched Ensembl release 105 using the keyword “major histocompatibility complex” to identify any additional gene models with homology with core MHC genes. Using identified core MHC genes from green anole and tuatara, we manually identified orthologous gene models in other squamate genomes included in Ensembl release 105, which includes four elapid snakes (blue-lipped sea krait [*Laticauda laticaudata*], Indian cobra [*Naja naja*], tiger snake [*Notechis scutatus*], and eastern brown snake [*Pseudonaja textilis*]) and two lizards (common wall lizard [*Podarcis muralis*] and Argentine black and white tegu [*Salvator merianae*]). For the brown anole we used tblastx to search the genome for MHC CDS sequences from all squamates, tuatara, human, mouse, chicken, and zebra finch. We retained all hits with percent identities of 70% or greater, lengths of 50 bp or greater, and e-values less than or equal to 1e-10. We cross-referenced these hits with gene models present in the brown anole gene annotation using bedtools to identify putative *mhc1* and *mhc2* orthologs.

We also identified genes contained on sequenced BAC clones for the green anole by first conducting keyword searches of gene models for the green anole, chicken, and human reference genomes on NCBI for ‘histocompatibility’, ‘hla’, ‘mhc’, ‘major histocompatibility’, ‘tap’, ‘atp’, ‘antigen transporter’. We downloaded any keyword results and conducted BLAST searches using our assembled BAC sequences. For each *A. carolinensis* query sequence, we aligned its top *A. carolinensis* match and calculated the number of different amino acids. If the similarity was greater than 99%, we assumed it to be the same locus; if the similarity was 95% – 99%, we assumed they were allelic variation at the same locus; and if the match was less than 95%, we assumed the genes were paralogous.

### **Identifying and assembling RNA-seq reads homologous to full length *mhc2β* in the green anole**

We investigated whether genome assembly artifacts may account for the putatively missing *mhc2β* homolog in the green anole and the gene annotation models lacking exon 2 in both species. We used BLAST to query both the green and brown anole genomes using the sequence for exon 2 retrieved from the single brown anole homolog where this exon was present (gene annotation ID ANOSAGT006753). No BLAST hits were observed in our search of the green anole genome and the only high-confidence hits in the brown anole genome were in the region with the annotated gene containing the original exon 2 sequence (i.e., self-hits). These results suggest that gene annotations for these two species are complete and high quality with respect to *mhc2β* homologs but that genome assembly artifacts may explain the lack of a second, full-length *mhc2β* homolog in the green anole. In other words, homologs lacking exon 2 were identified and properly annotated in both anole species but evidence of a complete *mhc2β* homolog was only observed in the brown anole, suggesting that this *mhc2β* homolog may not have been assembled well in the green anole genome. A related, less-probable hypothesis is that genome assembly artifacts, namely gaps, may be present in the regions surrounding one of the *mhc2β* homolog gene models in both species and that these gaps may correspond with a poor assembly in the regions that normally encode exon 2 in these species. However, for both species, we found no assembly gaps in the vicinity of gene models where exon 2 is missing, which strongly suggests that the gene annotation models lacking exon 2 features are not erroneous due to genome assembly artifacts in these regions of the genome.

To include a representative, full-length homolog of the green anole *mhc2β* in our evolutionary analyses, we turned to a large, diverse RNA-seq dataset from the Bgee database, which includes 47 datasets representing 16 tissue types (see above; Bastian et al., 2021). To identify RNA-seq reads with homology to *mhc2β*, we used BWA with the MEM algorithm and default options to map each Bgee dataset library against a reference sequence of the full length *mhc2β* transcript sequence obtained from the brown anole (gene annotation ID ANOSAGT006753). Mapped reads were extracted from the resulting BAM files using samtools v. 1.10 (Li et al., 2009; Li, 2011) and used to assemble transcripts using Trinity v. 2.10.0 (Grabherr et al., 2011; Haas et al., 2013) with default settings except the option --min_kmer_cov=1. We used BLAST searches against the NCBI NR database to identify transcripts with high homology to *mhc2β* records and incorporated one apparently full-length assembled transcript spanning exons 2 and 3 into our existing *mhc2β* multisequence alignment (see main text methods).

## **Supplementary Results**

### **BAC assemblies and gene annotations**

Between 2 and 25 contigs were assembled for each individual BAC clone. We removed 32 (15.4%) of our contigs from further analyses because they contained entirely or almost entirely transposable elements (TEs), consistent with the high percentage (~30%) of the anole genome composed of TEs (Alföldi et al., 2011; Tollis and Boissinot, 2011). Two BAC contigs (73F21_00014 and 73F21_00015) were duplicates of each other and were collapsed into a single contig. The remaining 48 contigs (59.2%) aligned well to 15 genomic scaffolds and two chromosomes (Supplementary Table 7; Supplementary Figure 3). A summary of the assembly and localization of contigs for each BAC library is as follows:

*BAC 73F21 -* BAC 73F21 assembled to 22 contigs 14 of which were retained. Six contigs aligned to scaffold Un_GL344142. The remaining eight each aligned with a different scaffold. Notably, contig 73F21_2 aligned to scaffold Un_AAWZ02036018 overlapping with contig 107B15_1.

*BAC 107B15 -* BAC 107B15 was assembled into two contigs. However, contig 107B15_1 (13,726 bp), was removed due to repetitive elements and failing to preferentially align to any one scaffold. Three portions of the remaining, contig 107B15_00001 each had acceptable alignments to different scaffolds: Un_AAWZ02036018, Un_GL343809, and Un_GL344142.

*BAC 118B8 -* BAC 118B8 assembled to ten contigs of which we retained five. We were unable to align any portion of this BAC with any of the other BACs sequenced in this study. However, contigs 118B8_00002 and 118B8_00003 aligned to both Un_GL344036 and Un_GL344134, and contig 118B8_00005 aligned uniquely to Un_AAWZ02038253.

*BAC 134P17 -* BAC 134P17 assembled to 22 contigs of which 13 were retained. Eleven of these contigs aligned to scaffold Un_GL343520. The remaining two, 134P17_00005, and 134P17_00018 each aligned best to a single scaffold, AAWZ02035787, and Un_GL343319 respectively.

*BAC 415L2 -* BAC 415L2 assembled to 25 contigs of which we retained 15. Of these 15 contigs, three aligned with Un_GL343676 and the remaining 12 with Un_GL343775. (See Supplementary Table 4 for all BLAT alignment information).

From our BAC contigs, we annotated a total of five *mhc2*, one *mhc1*, two *tap2*, and one *tap1*, and ten non-MHC related genes. Upon manual inspection the following loci discussed do not contain stop codons or frame shifts, and we therefore interpret them as real, coding genes. We sequenced two different *mhc2α* loci; one locus on 415L2_00003, and another single locus on the overlapping region of 73F21_2, and 107B15_00001. There is a single amino acid difference (0.4%) between 73F21_2/107B15_00001 and the best alignment match with predicted gene XP_016854554.1 which is likely due to allelic variation. The *mhc2α* gene on 415L2_00003 is 18.8% different between its best alignment match XP_003229154.3. Since our BAC and XP_003229154.3 BLAT align to different scaffolds we are comfortable in claiming that we have uncovered a previously unidentified *mhc2α* locus. We also identified a 28 amino acid sequence inserted into XP_003229154.3 which neither appears in either of our two BACs, nor in the Ensembl gene predicted for that region (ENSACAT00000011974.2). We conclude that there are a total of two *mhc2α* loci in the current *A. carolinensis* assembly plus a third (73F21_2) added from this research (Supplementary Table 8).

We sequenced through an *mhc2β-1* chain and *β-2* chain locus on BAC 134P17_00001. The amino acid sequence on the BAC is 11% different from the amino acid sequence in the best aligned predicted gene XP_008119271.1. The exon for the *mhc2β-1* domain is located in a large gap in the AnoCar2.0 assembly, part of which aligns with chromosome 3. We believe we have sequenced the full *mhc2β* gene on BAC 134P17_00001. Extensive *in silica* searching found two other predicted *mhc2* loci in the *A. carolinensis* genome, however neither of them align to our B-chain paralog, or other lizard orthologs. We conclude there are three *mhc2β* loci in the current *A. carolinensis* assembly (Supplementary Table 8) plus a fourth (from 134P17_00001) added from this research.

We sequenced through all twelve exons of the MHC class I related *tap2* gene. Exons 1 – 7 were found on contig 118B8_00001, and exons 8 – 12 were found on 118B8_00003. There is less than 1% difference between our sequence and the predicted gene in the green anole assembly (XP_008122042.1) strongly suggesting it is the same gene. This is the only locus of a *tap2* gene we were able to identify in our search of the *A. carolinensis* assembly. We also sequenced part of a *tap1* gene, which is also part of the MHC class I domain. This gene, located on BAC 118B8_00004, has a 2.3% amino acid difference between the most aligned predicted gene (XM_016998771.1) suggesting allelic variation. We were able to discern only one *tap1* locus on the current *A. carolinensis* genome assembly.

Finally, we sequenced an *mhc1* gene on BAC 118B8_03. The amino acid sequence is 4.9% different from its best aligned predicted gene (XP_008122234.2) suggesting allelic variation at this locus (chrUn_GL344134). In addition to this locus, our key word and sequence searches found 20 putative loci for *mhc1* genes. However, upon close inspection, four of these had identical sequences, another three shared different identical sequences, and two others shared a third identical sequence. We conclude that there are 15 *mhc1* loci in the current *A. carolinensis* genome assembly (Supplementary Table 8).

### **Anole-specific gene losses in the MHC**

Individual anole species were lacking certain genes. In the brown anole, several genes appeared to be missing, including *C6orf136*, *LSM2*, *MOG*, *MRPS18B*, and *SAPCD1*. Several distinct genes were missing from the green anole MHC region: *C2*, *C4A*, *DAXX*, *LST1*, *HSD17B8*, *LY6G6C*, *MPIG6B*, *NOTCH4*, *POU5F1*, *PRRC2A*, *RNF39*, *SLC39A7*, *TAPBP*, and *WDR46* (Figure 4). Aside from the signal of genes involved with the innate immunity complement system (i.e., *C4B* potentially missing in both anole species and *C2* and *C4A* that is absent in the green anole genome), these genes are involved in a range of physiological processes related to immunity, development, and other important biological functions in vertebrates. However, some missing genes in either anole species are known to cluster together in the human MHC (e.g., *LTA* and *LTB* in both species; *C6orf136* and *MRPS18B* in the brown anole; and *LTA*–*LTB*–*LST1*–*PRRC2A*, *LY6G6C*–*MPIG6B*, and *C2*–*C4A*–*C4B* in the green anole), which suggests that incomplete assembly or annotation of the anole genomes may account for these results, especially in the green anole.

### **Repeat content in *Anolis* MHC regions**

For the green anole, the MHC region had consistently high numbers of DNA transposons and LINE retrotransposons, which made up approximately 20% of windows across the region. Generally, less than 10% of any window was composed of other major repeat element groups, though there were some 100 kb windows where repeat families exceeded 10% (Figure 5). These patterns were similar in the MHC region of the brown anole but some noticeable differences emerged. First, while DNA and LINE elements were in roughly equal proportions in windows in the green anole, LINE elements were consistently more plentiful across 100 kb windows in the brown anole MHC region, which suggests alternative patterns of repeat proliferation between the two anole species. Moreover, the percentage of windows annotated as LINE elements grew across the MHC region of the brown anole, especially for windows beyond the 300 Mb coordinate. This growth in the abundance of LINE elements in this subregion of the brown anole genome appeared to be associated with a relative depletion of DNA transposons, which showed abundances of approximately 10% of less, which is more similar to the rest of the annotated repeat groups (Figure 5).

We also evaluated patterns of repeat composition in MHC and non-MHC regions of the six amniote species. All species, including the non-avian reptiles that are less represented in existing repeat databases, had a small number of unknown repeat annotations, indicating that our repeat identification methodology is high quality. As expected, both avian species had low levels of repeat element content genome-wide and in MHC regions, and squamate genomes and MHC regions more closely resemble the genomes and MHC regions of human and tuatara. However, lineage-specific patterns of high repeat abundance were evident for certain taxa and repeat classifications. For example, human genomes and MHC regions contain relatively greater amounts of SINE elements, which are lower in copy number in all other species.

### **Evolution and selection of anole *mhc1* homologs**

Based on the FEL analysis of *mhc1* homologs, where, contrary to *mhc2β*, sample sizes were sufficient to confidently report patterns of selection, 136 and 102 codons were invariable in the green and brown anole, respectively (Figure 10; Supplementary Table 14). Ninety-seven *mhc1* codons were detected as evolving neutrally in the green anole and 124 codons showed evidence of neutral evolution in the brown anole. We detected fewer codons with evidence of purifying selection in the *mhc1* datasets, including 41 and 46 codons under purifying selection in the green anole and brown anole, respectively, of which 19 codons were shared between the species. Both idiosyncratic and shared patterns of natural selection are evident between the green and brown anole across codons. In many cases, codons with different classifications between species are subject to purifying selection in one species and are invariable or evolving neutrally in the other species (e.g., codons 37, 57, 61, 62, 86, 153, and 160). There are a small number of selected peptide binding codons with the same classifications of natural selection between species (N = 4; codons 68, 69, 72, and 118) or with evidence of purifying selection in one species and diversifying selection in the other species (N = 1; codon 100; Supplementary Figure 13).

##

## **Literature Cited**

Alföldi, J., Di Palma, F., Grabherr, M., Williams, C., Kong, L., Mauceli, E., et al. (2011). The genome of the green anole lizard and a comparative analysis with birds and mammals. *Nature* 477, 587–591. doi: 10.1038/nature10390.

Altschul, S. F., Gish, W., Miller, W., Myers, E. W., and Lipman, D. J. (1990). Basic local alignment search tool. *J. Mol. Biol.* 215, 403–410. doi: 10.1016/S0022-2836(05)80360-2.

Armstrong, J., Hickey, G., Diekhans, M., Fiddes, I. T., Novak, A. M., Deran, A., et al. (2020). Progressive Cactus is a multiple-genome aligner for the thousand-genome era. *Nature* 587, 246–251. doi: 10.1038/s41586-020-2871-y.

Bao, W., Kojima, K. K., and Kohany, O. (2015). Repbase Update, a database of repetitive elements in eukaryotic genomes. *Mob. DNA* 6, 11. doi: 10.1186/s13100-015-0041-9.

Bárdos, J. I., Saurin, A. J., Tissot, C., Duprez, E., and Freemont, P. S. (2000). HPC3 Is a New Human Polycomb Orthologue That Interacts and Associates with RING1 and Bmi1 and Has Transcriptional Repression Properties. *J. Biol. Chem.* 275, 28785–28792. doi: 10.1074/jbc.M001835200.

Bastian, F. B., Roux, J., Niknejad, A., Comte, A., Fonseca Costa, S. S., de Farias, T. M., et al. (2021). The Bgee suite: integrated curated expression atlas and comparative transcriptomics in animals. *Nucleic Acids Res.* 49, D831–D847. doi: 10.1093/nar/gkaa793.

Bickel, P. E., Scherer, P. E., Schnitzer, J. E., Oh, P., Lisanti, M. P., and Lodish, H. F. (1997). Flotillin and Epidermal Surface Antigen Define a New Family of Caveolae-associated Integral Membrane Proteins. *J. Biol. Chem.* 272, 13793–13802. doi: 10.1074/jbc.272.21.13793.

Bristow, J., Tee, M., Gitelman, S., Mellon, S., and Miller, W. (1993). Tenascin-X: a novel extracellular matrix protein encoded by the human XB gene overlapping P450c21B. *J. Cell Biol.* 122, 265–278. doi: 10.1083/jcb.122.1.265.

Burge, C., and Karlin, S. (1997). Prediction of complete gene structures in human genomic DNA. *J. Mol. Biol.* 268, 78–94. doi: 10.1006/jmbi.1997.0951.

Fitzgibbon, J., Gillett, G. T., Woodward, K. J., Boyle, J. M., Wolfe, J., and Povey, S. (1993). Mapping of RXRB to human chromosome 6p21. 3. *Ann. Hum. Genet.* 57, 203–209. doi: 10.1111/j.1469-1809.1993.tb01596.x.

Flynn, J. M., Hubley, R., Goubert, C., Rosen, J., Clark, A. G., Feschotte, C., et al. (2020). RepeatModeler2 for automated genomic discovery of transposable element families. *Proc. Natl. Acad. Sci.* 117, 9451–9457. doi: 10.1073/pnas.1921046117.

Gaczynska, M., Rock, K. L., Spies, T., and Goldberg, A. L. (1994). Peptidase activities of proteasomes are differentially regulated by the major histocompatibility complex-encoded genes for LMP2 and LMP7. *Proc. Natl. Acad. Sci.* 91, 9213–9217. doi: 10.1073/pnas.91.20.9213.

Gordon, A., and Hannon, G. (2010). *Fastx-toolkit*.

Grabherr, M. G., Haas, B. J., Yassour, M., Levin, J. Z., Thompson, D. A., Amit, I., et al. (2011). Full-length transcriptome assembly from RNA-Seq data without a reference genome. *Nat. Biotechnol.* 29, 644–652. doi: 10.1038/nbt.1883.

Haas, B. J., Papanicolaou, A., Yassour, M., Grabherr, M., Blood, P. D., Bowden, J., et al. (2013). De novo transcript sequence reconstruction from RNA-seq using the Trinity platform for reference generation and analysis. *Nat. Protoc.* 8, 1494–1512. doi: 10.1038/nprot.2013.084.

Holt, C., and Yandell, M. (2011). MAKER2: an annotation pipeline and genome-database management tool for second-generation genome projects. *BMC Bioinformatics* 12, 491. doi: 10.1186/1471-2105-12-491.

Howe, K. L., Achuthan, P., Allen, J., Allen, J., Alvarez-Jarreta, J., Amode, M. R., et al. (2021). Ensembl 2021. *Nucleic Acids Res.* 49, D884–D891. doi: 10.1093/nar/gkaa942.

Janeway Jr., C. A., Travers, P., Walport, M., and Shlomchik, M. J. (2001). “The major histocompatibility complex and its functions,” in *Immunobiology: The Immune System in Health and Disease* (New York: Garland Science). Available at: https://www.ncbi.nlm.nih.gov/books/NBK27156/ [Accessed June 10, 2022].

Jurka, J., Kapitonov, V. V., Pavlicek, A., Klonowski, P., Kohany, O., and Walichiewicz, J. (2005). Repbase Update, a database of eukaryotic repetitive elements. *Cytogenet. Genome Res.* 110, 462–467. doi: 10.1159/000084979.

Kelley, J., Walter, L., and Trowsdale, J. (2005). Comparative genomics of major histocompatibility complexes. *Immunogenetics* 56, 683–695. doi: 10.1007/s00251-004-0717-7.

Kent, W. J. (2002). BLAT—The BLAST-Like Alignment Tool. *Genome Res.* 12, 656–664. doi: [10.1101/gr.229202](https://doi.org/10.1101/gr.229202).

Koch, M., Camp, S., Collen, T., Avila, D., Salomonsen, J., Wallny, H.-J., et al. (2007). Structures of an MHC Class I Molecule from B21 Chickens Illustrate Promiscuous Peptide Binding. *Immunity* 27, 885–899. doi: 10.1016/j.immuni.2007.11.007.

Kusumi, K., Kulathinal, R. J., Abzhanov, A., Boissinot, S., Crawford, N. G., Faircloth, B. C., et al. (2011). Developing a community-based genetic nomenclature for anole lizards. *BMC Genomics* 12, 554. doi: 10.1186/1471-2164-12-554.

Li, H. (2011). A statistical framework for SNP calling, mutation discovery, association mapping and population genetical parameter estimation from sequencing data. *Bioinformatics* 27, 2987–2993. doi: 10.1093/bioinformatics/btr509.

Li, H., and Durbin, R. (2009). Fast and accurate short read alignment with Burrows–Wheeler transform. *Bioinformatics* 25, 1754–1760. doi: 10.1093/bioinformatics/btp324.

Li, H., Handsaker, B., Wysoker, A., Fennell, T., Ruan, J., Homer, N., et al. (2009). The Sequence Alignment/Map format and SAMtools. *Bioinformatics* 25, 2078–2079. doi: 10.1093/bioinformatics/btp352.

Li, X., Zhang, L., Liu, Y., Ma, L., Zhang, N., and Xia, C. (2020). Structures of the MHC-I molecule BF2*1501 disclose the preferred presentation of an H5N1 virus-derived epitope. *J. Biol. Chem.* 295, 5292–5306. doi: 10.1074/jbc.RA120.012713.

Manni, M., Berkeley, M. R., Seppey, M., Simão, F. A., and Zdobnov, E. M. (2021). BUSCO Update: Novel and Streamlined Workflows along with Broader and Deeper Phylogenetic Coverage for Scoring of Eukaryotic, Prokaryotic, and Viral Genomes. *Molecular Biology and Evolution* 38, 4647–4654. doi: [10.1093/molbev/msab199](https://doi.org/10.1093/molbev/msab199).

Mapleson, D., Garcia Accinelli, G., Kettleborough, G., Wright, J., and Clavijo, B. J. (2017). KAT: a K-mer analysis toolkit to quality control NGS datasets and genome assemblies. *Bioinformatics* 33, 574–576. doi: [10.1093/bioinformatics/btw663](https://doi.org/10.1093/bioinformatics/btw663).

Mourad, W. A., Kfoury, H. K., and Ali, M. A. (1997). B- and T-Lymphocyte Distribution in Benign and Malignant Lymphoepithelial Lesions of the Parotid Gland: Correlation with Epstein-Barr Virus Expression and a Proposed Mechanism of Malignant Transformation. *Ann. Saudi Med.* 17, 4–9. doi: 10.5144/0256-4947.1997.4.

Ondov, B. D., Treangen, T. J., Melsted, P., Mallonee, A. B., Bergman, N. H., Koren, S., et al. (2016). Mash: fast genome and metagenome distance estimation using MinHash. *Genome Biol.* 17, 132. doi: 10.1186/s13059-016-0997-x.

Rhodes, D. A., Stammers, M., Malcherek, G., Beck, S., and Trowsdale, J. (2001). The Cluster of BTN Genes in the Extended Major Histocompatibility Complex. *Genomics* 71, 351–362. doi: 10.1006/geno.2000.6406.

Saper, M. A., Bjorkman, P. J., and Wiley, D. C. (1991). Refined structure of the human histocompatibility antigen HLA-A2 at 2.6 Å resolution. *J. Mol. Biol.* 219, 277–319. doi: 10.1016/0022-2836(91)90567-P.

Smit, A. F. A., Hubley, R., and Green, P. (2013). *RepeatMasker Open-4.0*. Available at: http://repeatmasker.org/.

Stanke, M., Steinkamp, R., Waack, S., and Morgenstern, B. (2004). AUGUSTUS: a web server for gene finding in eukaryotes. *Nucleic Acids Res.* 32, W309–W312. doi: 10.1093/nar/gkh379.

The MHC sequencing consortium (1999). Complete sequence and gene map of a human major histocompatibility complex. *Nature* 401, 921–923. doi: 10.1038/44853.

Tollis, M., and Boissinot, S. (2011). The transposable element profile of the *Anolis* genome. *Mob. Genet. Elem.* 1, 107–111. doi: 10.4161/mge.1.2.17733.

Wang, Y., Qu, Z., Ma, L., Wei, X., Zhang, N., Zhang, B., et al. (2021). The Crystal Structure of the MHC Class I (MHC-I) Molecule in the Green Anole Lizard Demonstrates the Unique MHC-I System in Reptiles. *J. Immunol.* 206, 1653–1667. doi: 10.4049/jimmunol.2000992.

Zhang, S. V., Zhuo, L., and Hahn, M. W. (2016). AGOUTI: improving genome assembly and annotation using transcriptome data. *GigaScience* 5, 31. doi: 10.1186/s13742-016-0136-3.

Zhang, S.-H., Wang, C.-J., Shi, L., Li, X.-H., Zhou, J., Song, L.-B., et al. (2013). High Expression of FLOT1 Is Associated with Progression and Poor Prognosis in Hepatocellular Carcinoma. *PLOS ONE* 8, e64709. doi: 10.1371/journal.pone.0064709.

##

## **Supplementary Figures**


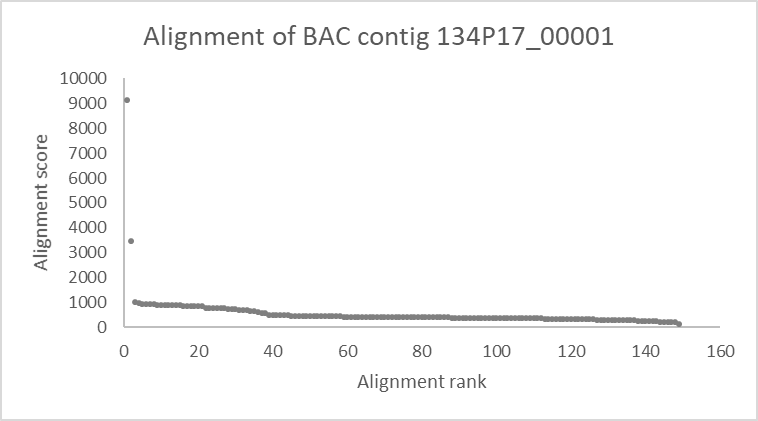

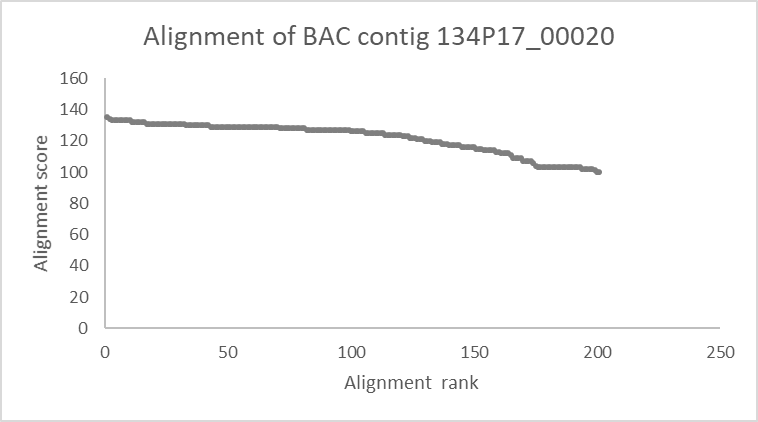


**b.**

**a.**

**Supplementary Figure 1.** Exemplary graphs of BAC contigs BLAT-aligned to the *Anolis carolinensis* genome rank-ordered by alignment score. **A.** An example of a clear best contig alignment. This contig was retained for annotation and genome construction with two alignments to be manually curated. **B**. An example of a contig with ambiguous alignment. This contig was discarded from further analyses.


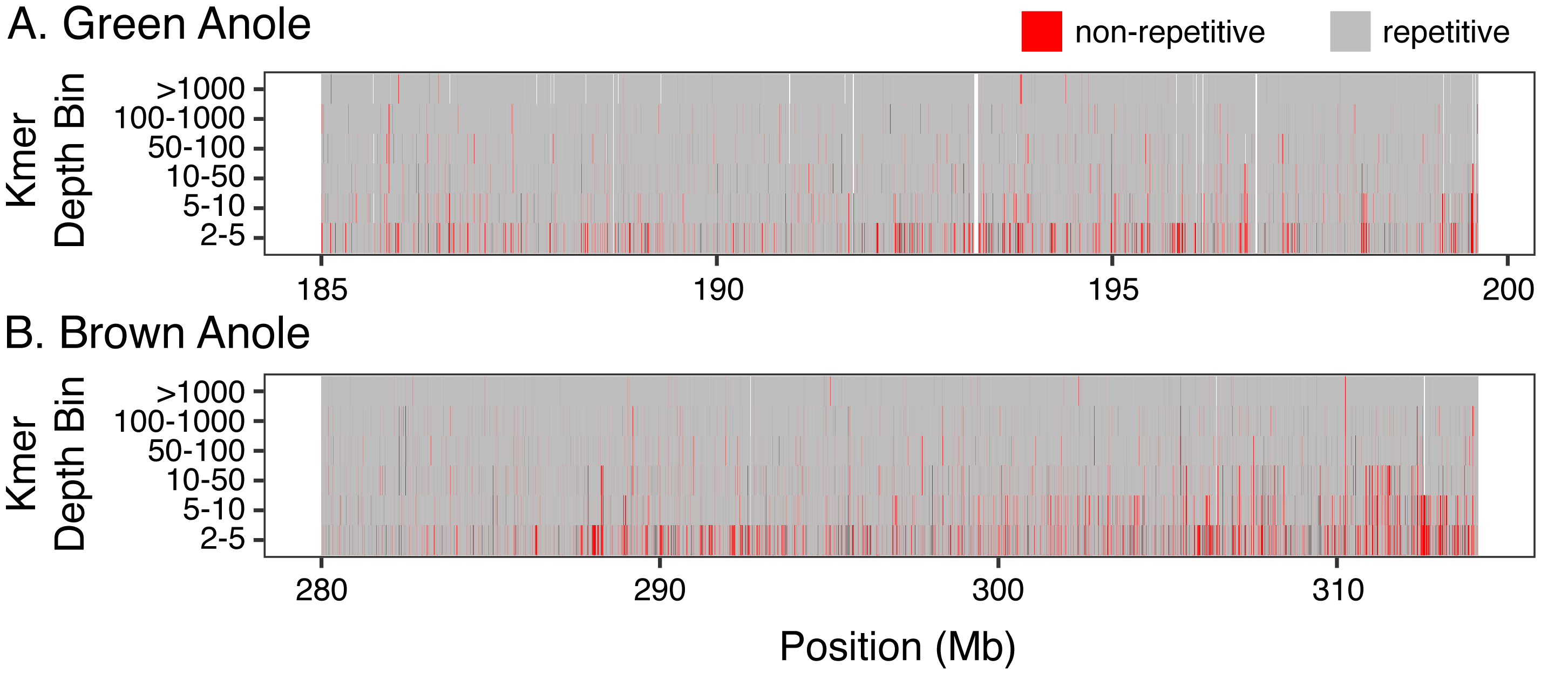


**Supplementary Figure 2.** Summary of genome assembly quality of the MHC regions of (**A**) green anole chromosome 2 and (**B**) brown anole scaffold_2 based on the depth of 27-mers. The position of the beginning of each k-mer is indicated and cells are color coded based on whether the k-mer coordinate is annotated as repetitive based on our repeat annotation.


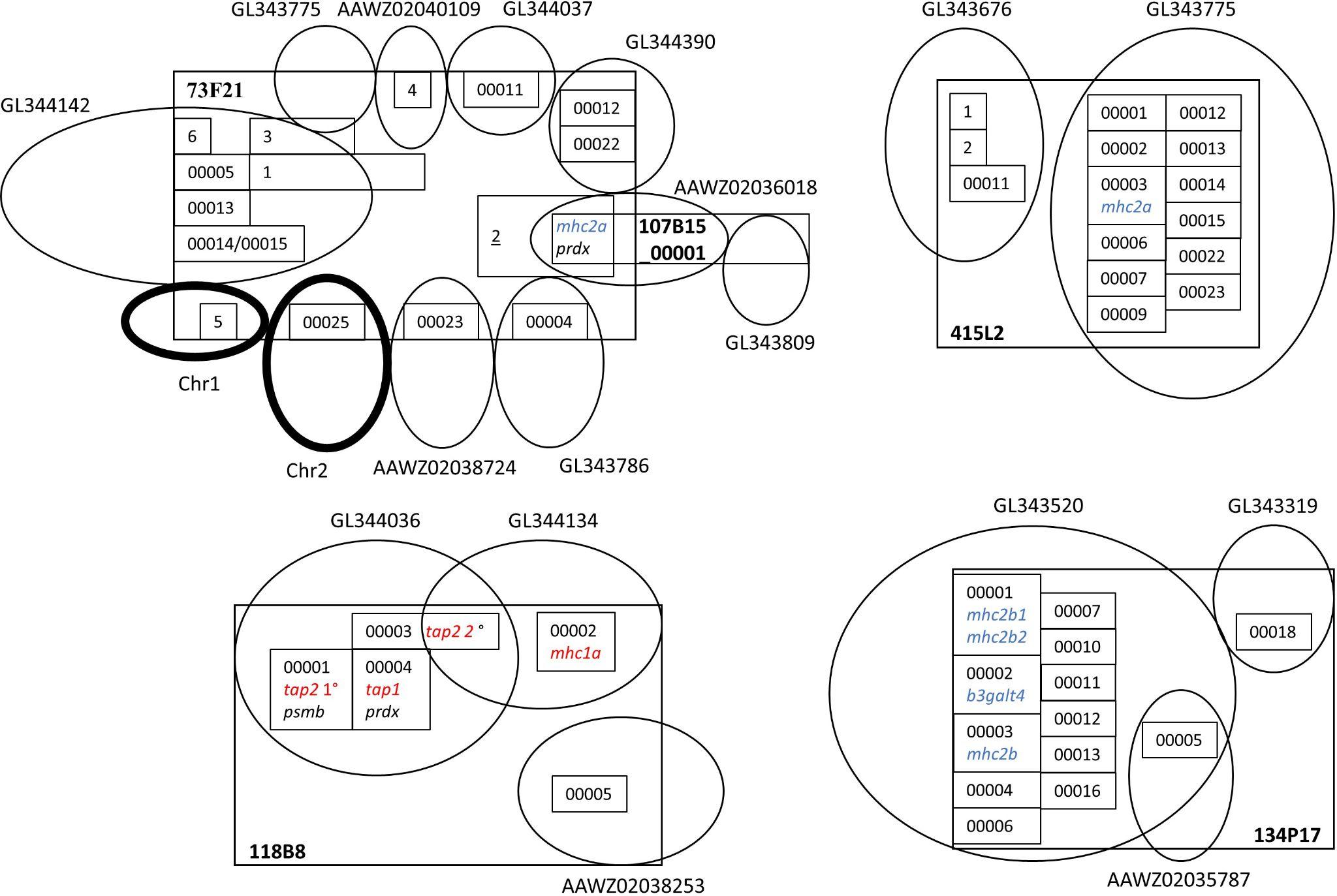


**Supplementary Figure 3.** Assembly and annotation of four *Anolis carolinensis* BAC contigs. BAC elements are rectangular, with large, central rectangles representing the BAC and smaller rectangles within representing relative locations of the BAC contigs. Genomic scaffolds are ovals and chromosomes are ovals with heavy lines. Annotated genes are noted by gene symbol where red indicates MHC class I and blue indicates MHC class II region genes. Note that BAC 107B15 assembled to a single contig (00001) and some contigs span multiple scaffolds. This representation is intentional as the two ends of these contigs aligned to different scaffolds. Genes with a degree symbol (°) indicate different exons of the same gene. When possible, contigs are arranged in numeric order. This is simply for aesthetic purposes and should not be interpreted as an argument for the linear order of the BAC sequence.


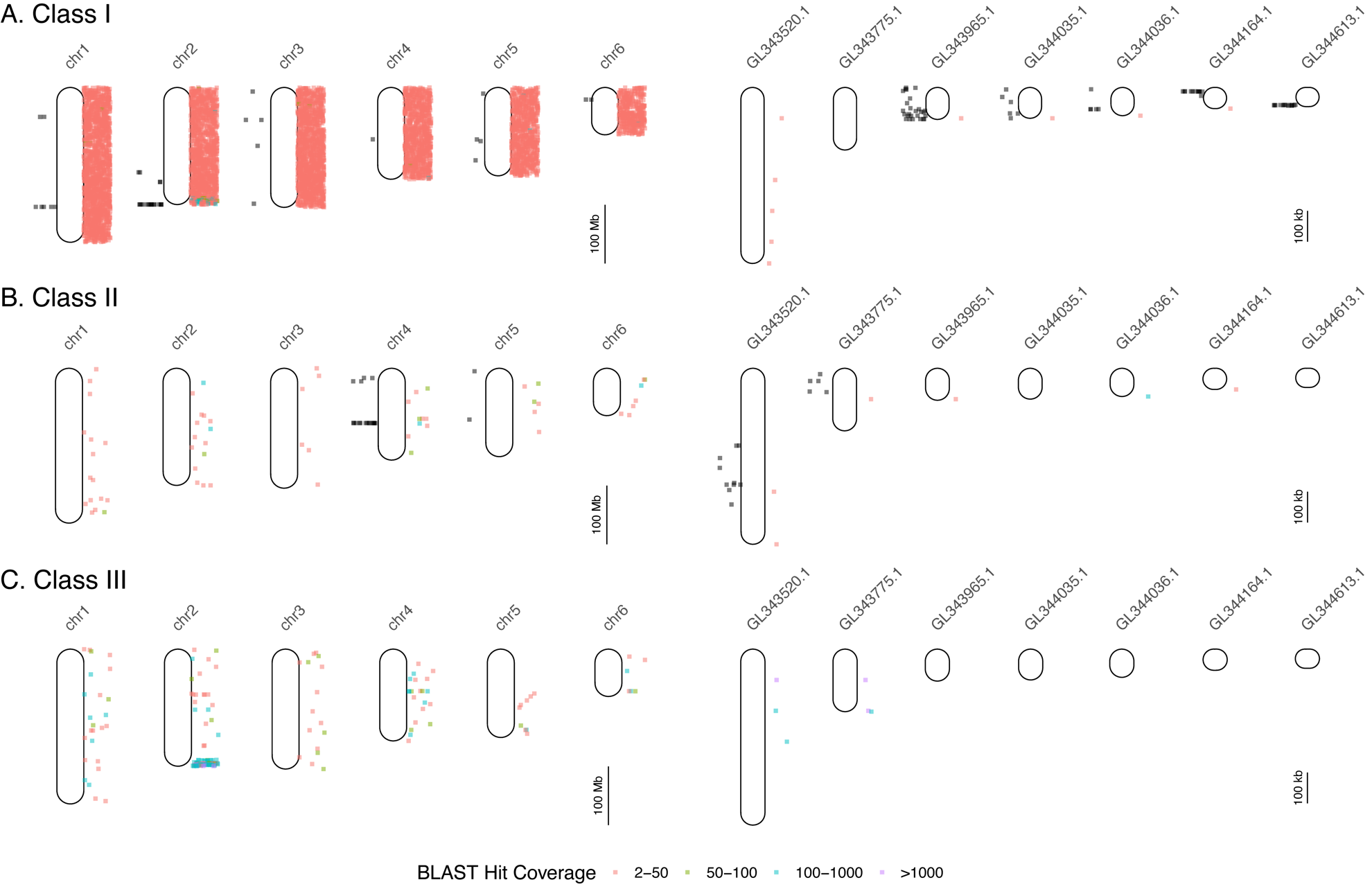


**Supplementary Figure 4.** Results of mapping of BAC end sequences (BESs) of BACs detected in hybridizations and BLAST searches of squamate MHC protein data in the green anole genome. Each point indicates the locations of mapped BESs (black; left of chromosome maps) or BLAST hits (binned by hit coverage; right of chromosome maps). Results are displayed separately for class I (**A**), class II (**B**), and class III (**C**) genes for all six chromosomes and for unplaced scaffolds with five or more mapped BESs. Note differences in scale between panels the panels devoted to chromosomes (left column) and unplaced scaffolds (right column). See Supplementary Table 6 for the results of the BLAST mapping of BESs.


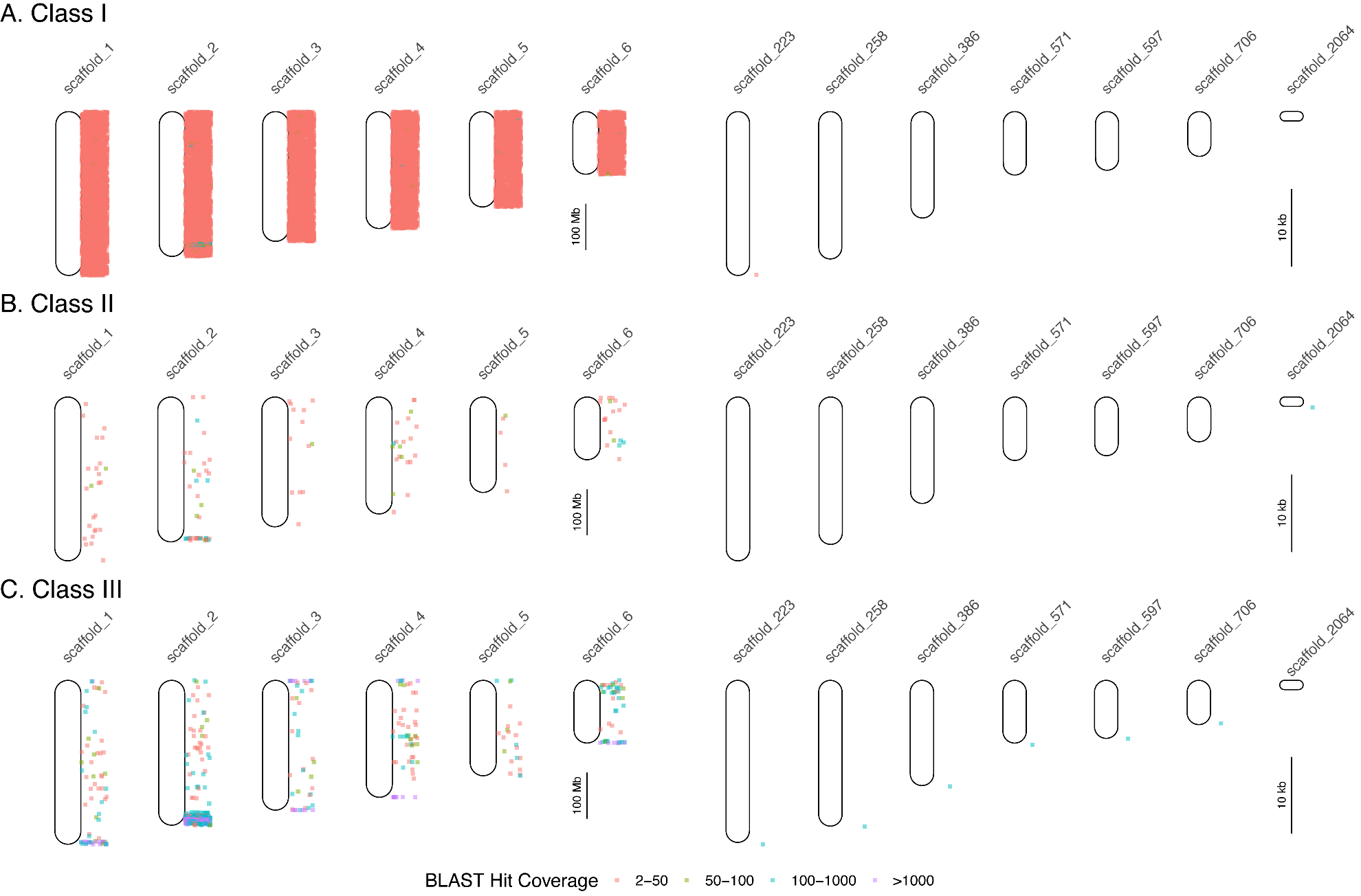


**Supplementary Figure 5.** Results of BLAST searches of squamate MHC protein data in the brown anole genome. Each point indicates the locations of BLAST hits (binned by hit coverage; right of chromosome maps). Results are displayed separately for class I (**A**), class II (**B**), and class III (**C**) genes for all six scaffolds, which correspond to the six macrochromosomes, and for unplaced scaffolds with at least 100 hits and average hits per 100 bp of greater than 2. Note differences in scale between panels the panels devoted to chromosomes (left column) and unplaced scaffolds (right column).


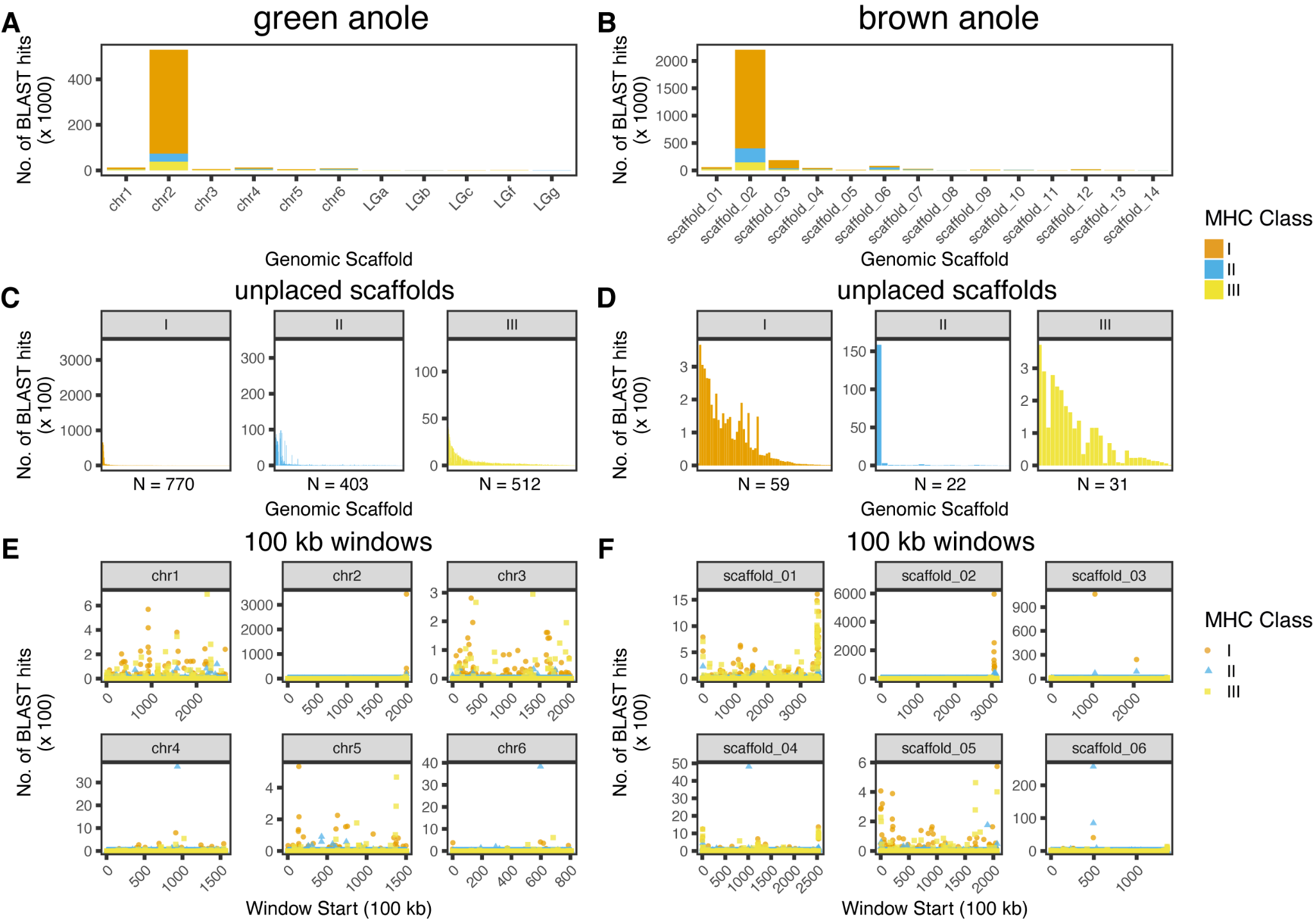


**Supplementary Figure 6.** Results of BLAST-based genomic location of MHC homology based on NCBI records for human or mouse for green anole (left column) and brown anole (right column). **A.** Number of BLAST hits per MHC class across chromosomes in the green anole genome assembly. **B.** Number of BLAST hits per MHC class across macro-scaffolds in the brown anole genome assembly. **C.** Number of BLAST hits per MHC class across unplaced scaffolds in the green anole genome. **D.** Number of BLAST hits per MHC class across micro-scaffolds in the brown anole genome assembly. **E.** Number of BLAST hits per MHC class across 100 kb windows in scaffolds longer than 1 Mb in the green anole genome. **F.** Number of BLAST hits per MHC class across 100 kb windows in scaffolds longer than 1 Mb in the brown anole genome.


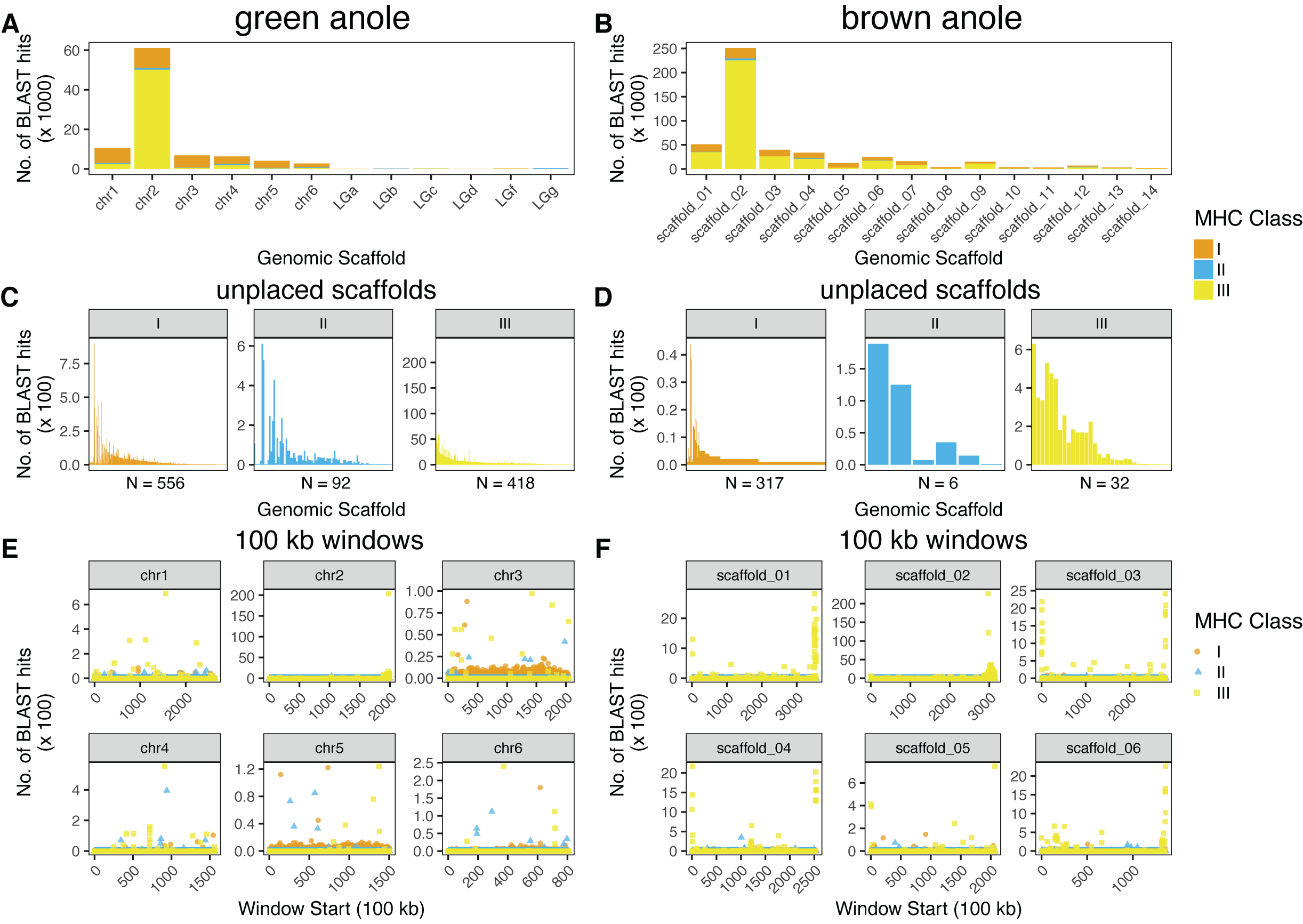


**Supplementary Figure 7.** Results of BLAST-identified genomic locations of MHC homology based on NCBI records for Squamata for green anole (left column) and brown anole (right column). Number of BLAST hits per MHC class across chromosomes in the **A.** green anole and **B.** brown anole genome assemblies. **C.** Number of BLAST hits per MHC class across unplaced scaffolds in the **C.** green anole and **D.** brown anole genome assemblies. Number of BLAST hits per MHC class across 100 kb windows in scaffolds longer than 1 Mb in the **E.** green anole and **F.** brown anole genome assemblies.


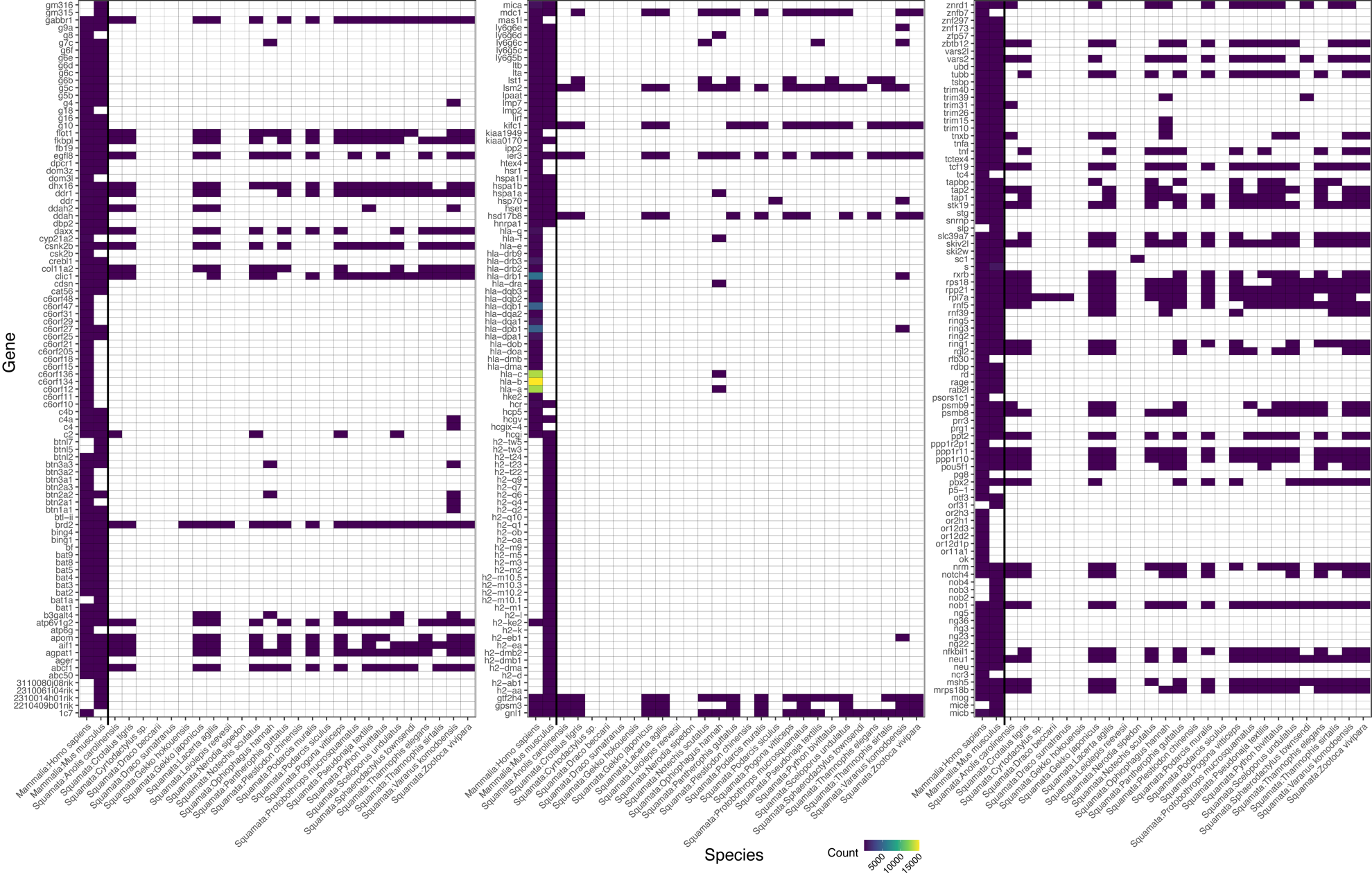


**Supplementary Figure 8.** Results of GenBank searches of 414 known MHC gene identifiers for human, mouse, and chicken from the literature. The number of resulting GenBank records for each gene ID is displayed for GenBank records from human, mouse, and any squamate species with relevant records. For each panel, GenBank results for human and mouse are located to the left of the bold, vertical line, while all records to the right of the line are from squamate species. The combined human and mouse protein dataset and the squamate protein dataset were used to search for homology against the green and brown anole genomes using BLAST.


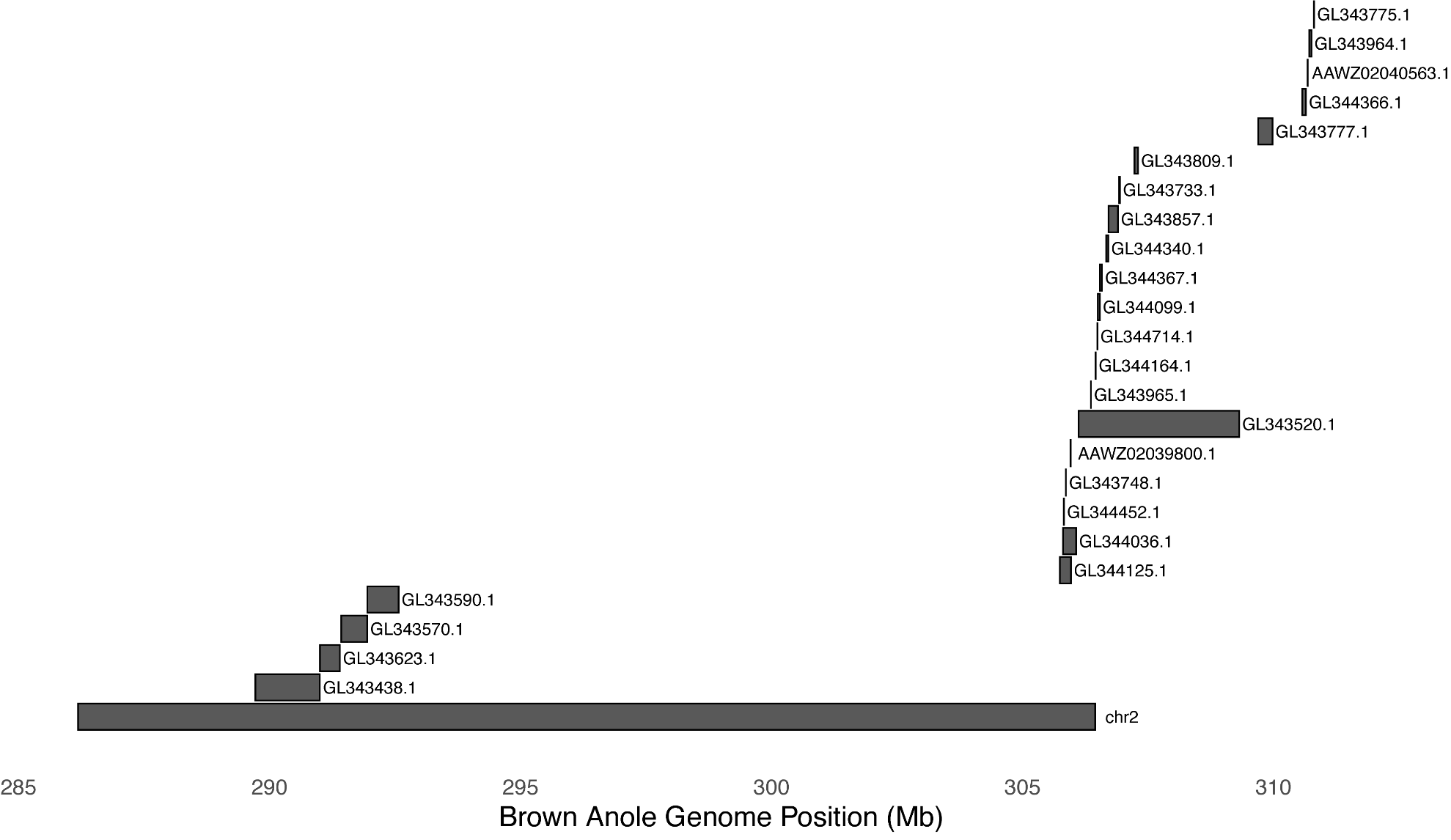


**Supplementary Figure 9.** Summary of the arrangement of green anole chromosomes and unplaced scaffolds based on a whole-genome alignment between the green and brown anole genomes. Coordinates along the x-axis indicate the position of each green anole scaffold based on the brown anole genome. Placement of each scaffold is based on evidence of synteny based on multiple co-linear alignment blocks between the anole genomes and is, therefore, approximate, which does not enable reliable interpretation of overlaps and gaps.

**
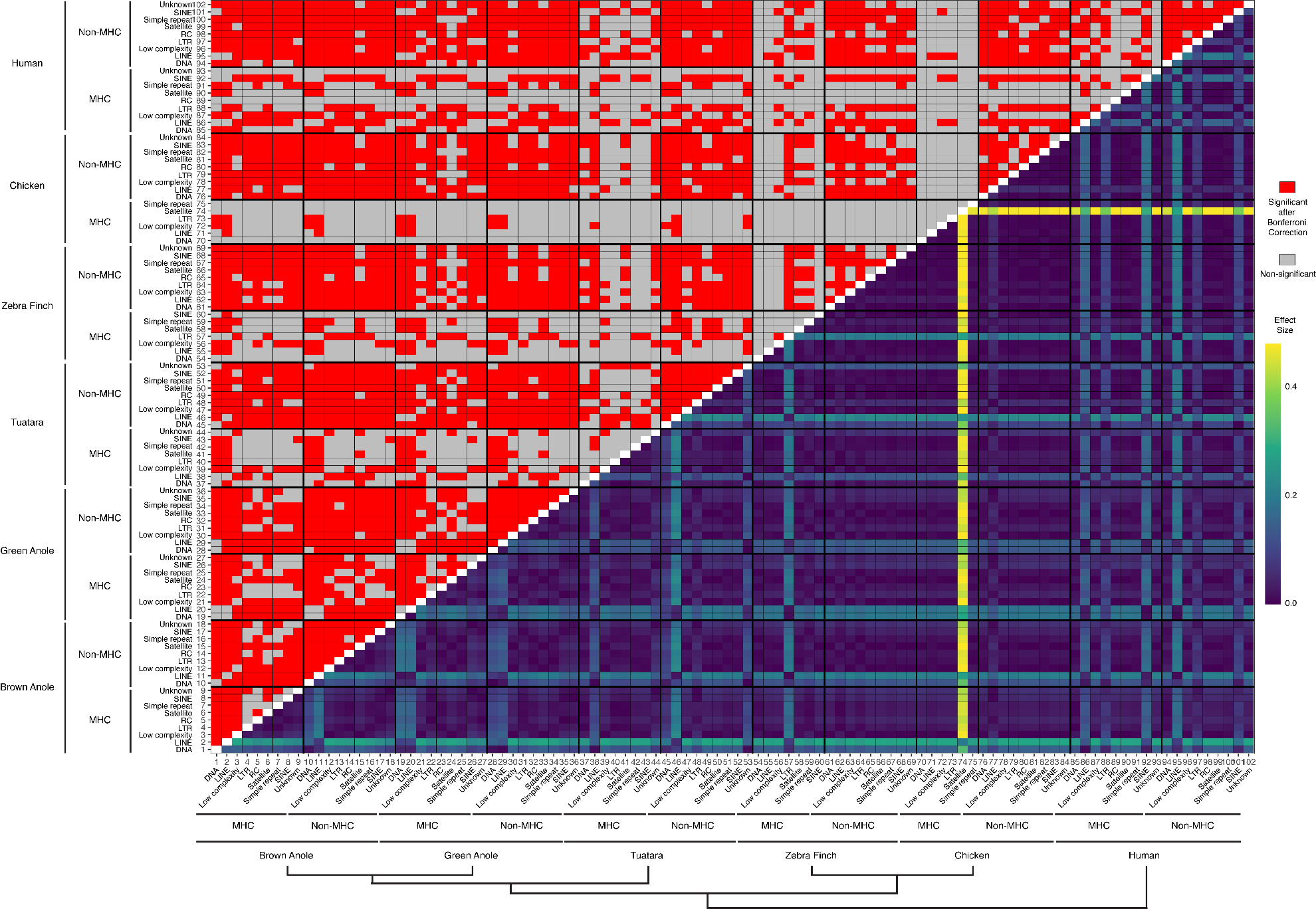
**

**Supplementary Figure 10.** Results of pairwise statistical comparisons between distributions of individual repeat classifications in MHC and non-MHC regions across six amniote species. Vertical and horizontal lines indicate the margins of MHC or non-MHC regions for each species. Significant pairwise comparisons were based Dunn tests with Bonferroni-corrected p-values calculated across all three panels in Figure 7 (N = 5,349 comparisons; p ≤ 0.05; yellow cells) and all pairwise comparisons for repeat element composition are summarized above the diagonal (N = 5,349 comparisons; p ≤ 0.05; red cells). Differences in the pairwise median measures of genomic composition (i.e., effect sizes) are summarized below the diagonal for each pairwise comparison. The color scales to the right indicates the effect sizes (proportions of repeats). See Supplementary Table 11 for the summary statistics underlying repeat proportion distributions and the results of pairwise statistical comparisons between each repeat classification, genomic region (MHC and non-MHC), and amniote species. Abbreviations for repeat elements are as follows: DNA = DNA transposons, LINE = Long Interspersed Nuclear Element; LTR = Long Terminal Repeat elements; RC = Rolling Circle elements; and SINE = Short Interspersed Nuclear Element.

**
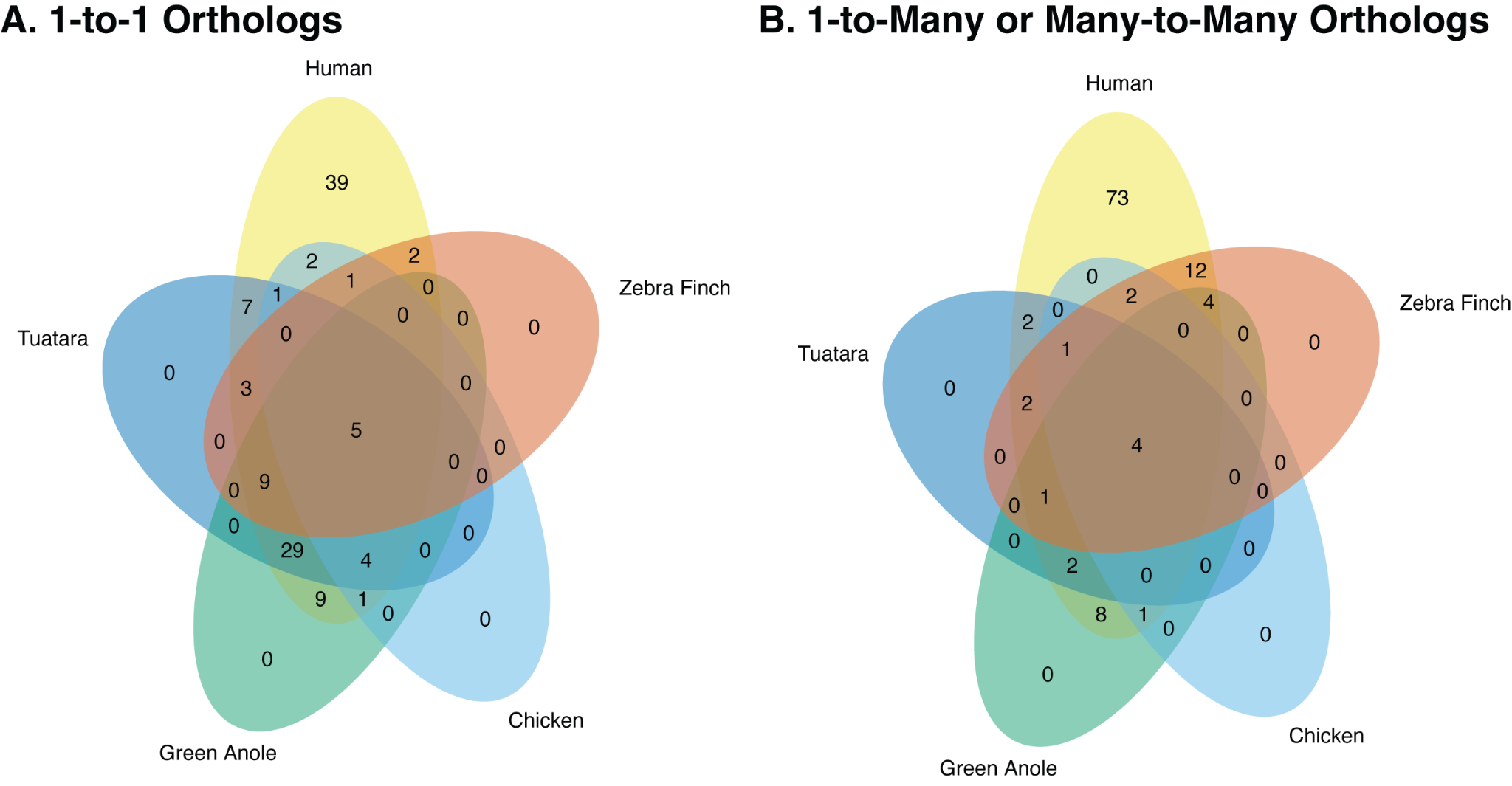
**

**Supplementary Figure 11.** Venn diagrams of **A.** 1-to-1 orthologs and **B.** 1-to-many or many-to-many orthologs across the five species included in the Ensembl comparative genomics database. See Supplementary Table 12 for the full details of gene orthology relationships between human and the other species.


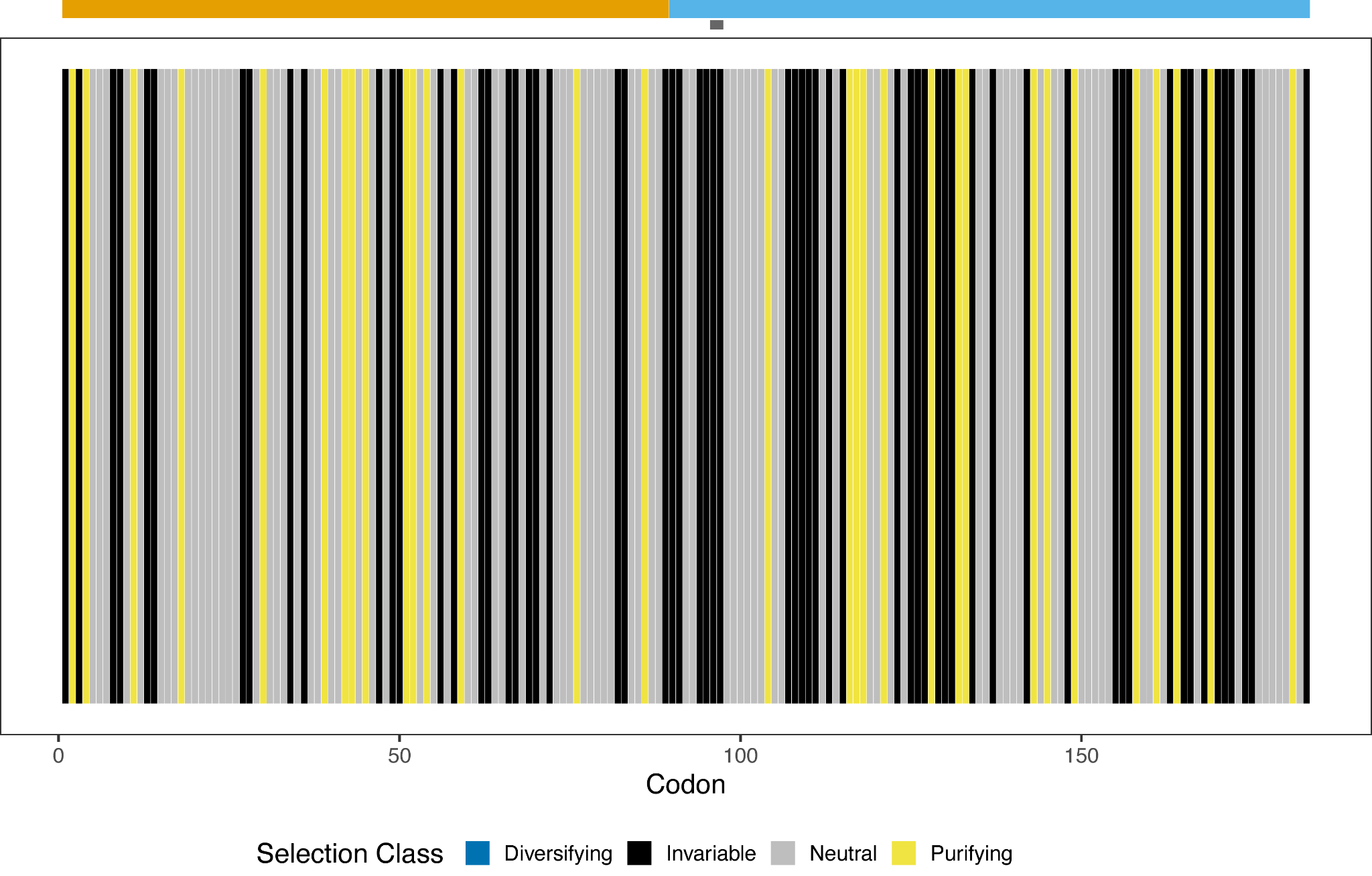


**Supplementary Figure 12.** Results of the fixed effect likelihood (FEL) analysis of the combined anole *mhc2β* dataset with an outgroup sequence from *Podarcis*. A vertical line represents each codon and is colored based on the inferred codon classification. The margins and span of exons 2 (orange) and 3 (blue) are indicated with bars over each panel and a two-amino acid insertion in exon 3 in *Podarcis* relative to *Anolis* is indicated with a grey bar small, leading to a total alignment length that is two amino acids longer (183 vs. 181 amino acids) than in the anole-specific *mhc2β* dataset summarized in the main text (see Figure 10E). See Supplementary Table 14 for the per-codon results of the FEL analysis of this dataset.


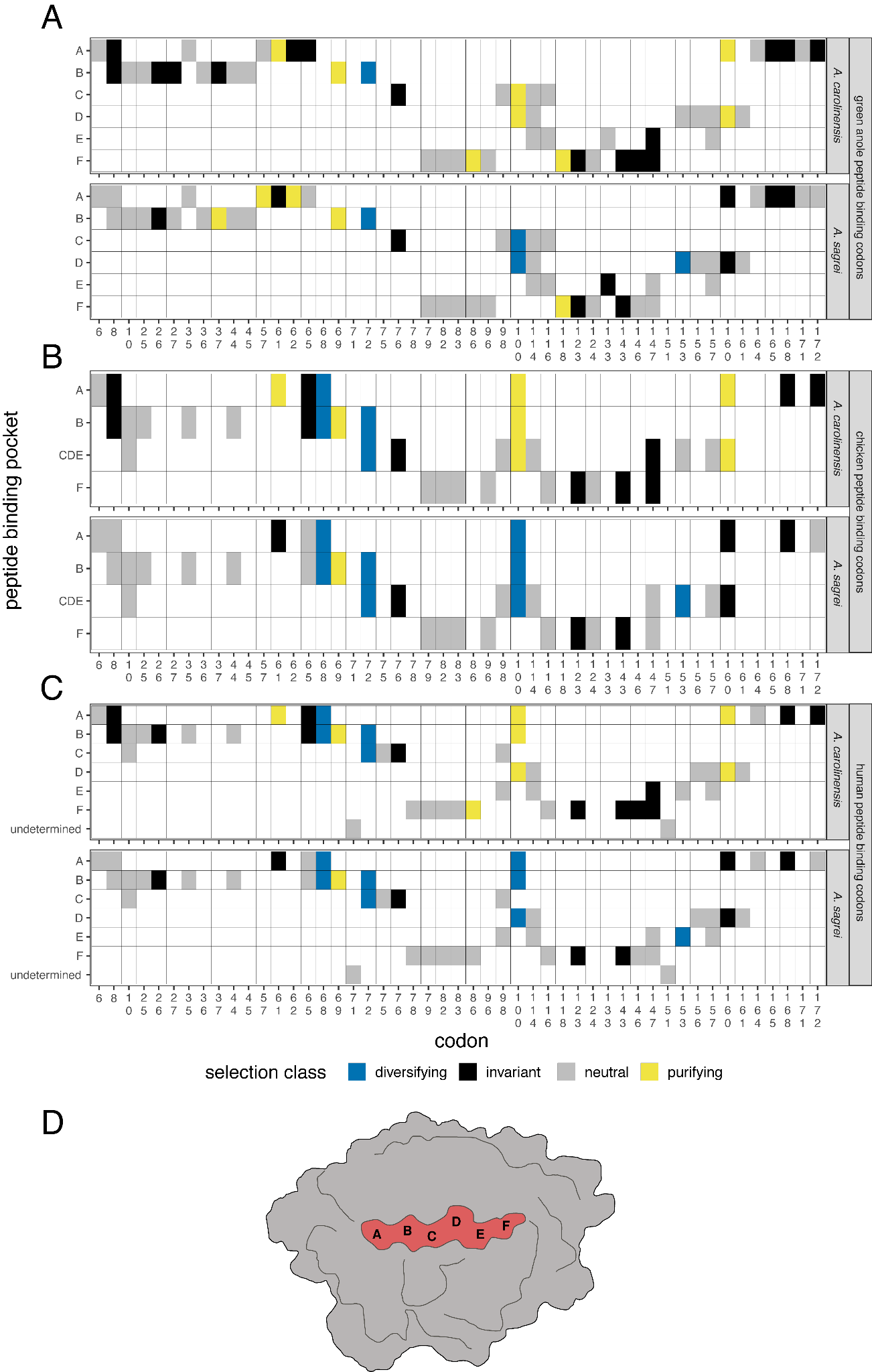


**Supplementary Figure 13.** Functional context of codon-specific selection of *mhc1* homologs from the green and brown anole based on known peptide binding codons of three amniote species: (**A**) green anole, (**B**) chicken, and (**C**) human. **D.** An illustration of the green anole *mhc1* protein with the location of each peptide binding pocket modified from Wang et al. (Wang et al., 2021). Known peptide binding codons are based on crystal structures of *mhc1* for green anole (Wang et al., 2021), chicken (Li et al., 2020), and human (Saper et al., 1991), with knowledge that chicken *mhc1* contains a large, central binding groove combining binding pockets C, D, and E (Koch et al., 2007), which form separate pockets in human and green anole. Uncolored (white) cells reflect codons that are not involved in peptide bonding in a given protein binding pocket. See Supplementary Table 14 for details of the known peptide binding codons and their position in the *mhc1* alignments of both anole species.
